# Synchronized Mascarene volcanism reveals 400 kyr cycles in melt supply from the Réunion plume

**DOI:** 10.1038/s41467-026-72855-1

**Published:** 2026-05-07

**Authors:** Vincent Famin, Xavier Quidelleur, Laurent Michon, Maëlis Arnould

**Affiliations:** 1 Université Paris Cité, Institut de Physique du Globe de Paris, CNRS, UMR 7154, 75238, Paris, cedex 05 France; 2 Université de La Réunion, Laboratoire GéoSciences Réunion, 97744, Saint-Denis, cedex 09 La Réunion France; 3 Université Paris-Saclay, CNRS, GEOPS, 91405 Orsay, France; 4 Université Clermont Auvergne, CNRS, IRD, OPGC, Laboratoire Magmas et Volcans, F-63000 Clermont-Ferrand, France; 5 Lyon 1 Université, ENSL, UJM, CNRS, UMR 5267, LGL-TPE, F-69622 Villeurbanne, France

**Keywords:** Stratigraphy, Volcanology, Geomorphology

## Abstract

Many hotspots display evidence of fluctuating magmatism through time, at periods of 1–20 Myr, reflecting variations in melt production and/or extraction within mantle plumes. Here we show that the Réunion hotspot exhibits ~10 times shorter fluctuations of magmatic activity. Using fieldwork, K-Ar geochronology, and geomorphology, we reconstruct the volcanic histories of Réunion and Mauritius, the youngest expressions of the Réunion hotspot. Our results reveal coeval phases of volcanic activity and repose on both islands over the past 3 Ma, with an average recurrence interval of 370 ± 202 kyr. Given the ~230 km separation between the islands and the heterogeneity of the underlying lithosphere, this large-scale volcanic synchronization is most consistent with quasi-periodic modulation of melt supply at mantle depths. The ~400 kyr timescale resembles the eruptive tempo of the Deccan Traps, suggesting that such short-period cyclicity may represent a long-lived characteristic of the Réunion hotspot.

## Abstract (web summary)

This study shows that volcanism at the Réunion hotspot fluctuates far more rapidly than previously thought, with synchronized activity cycles on Réunion and Mauritius every ~400,000 years, likely driven by periodic changes in deep mantle melt supply.

## Introduction

Oceanic island volcanoes are thought to be produced as convective plumes of hot mantle rise toward the Earth’s surface and undergo partial melting^1^. As lithospheric plates move over a mantle plume, new volcanoes form sequentially and build a hotspot chain in the direction of plate motion. Variations in the volumes of erupted and underplated magmas, and periods of reduced or absent activity, indicate that magma supply can fluctuate over hotspot lifetimes, likely in a periodic manner^2–4^. Such temporal variability provides constraints on plume dynamics and on the processes that regulate melt production and delivery. For instance, 5–10 Myr periodical magmatic pulses of the Iceland hotspot have been interpreted as “solitary waves”^5^ controlled by viscosity contrasts between the plume and the ambient mantle^6^. Many other plume mechanisms may generate fluctuations of magmatic activity in hotspots and large igneous provinces, including geochemical or thermal heterogeneities within plumes^7–9^, or instabilities generated by plume geometries^10–13^. The recognition of magmatic fluctuations in hotspots is therefore of great interest for understanding plume and mantle dynamics. However, most reconstructions of hotspot magma flux rely on age-distance relationships along hotspot trails and typically resolve variations at ≥1 Myr, comparable to the lifetime of individual volcanic edifices^2–4,14^. Shorter-period magmatic fluctuations may therefore remain undetected, yet could provide additional constraints on melt transport and plume–lithosphere interactions.

**Fig. 1:**
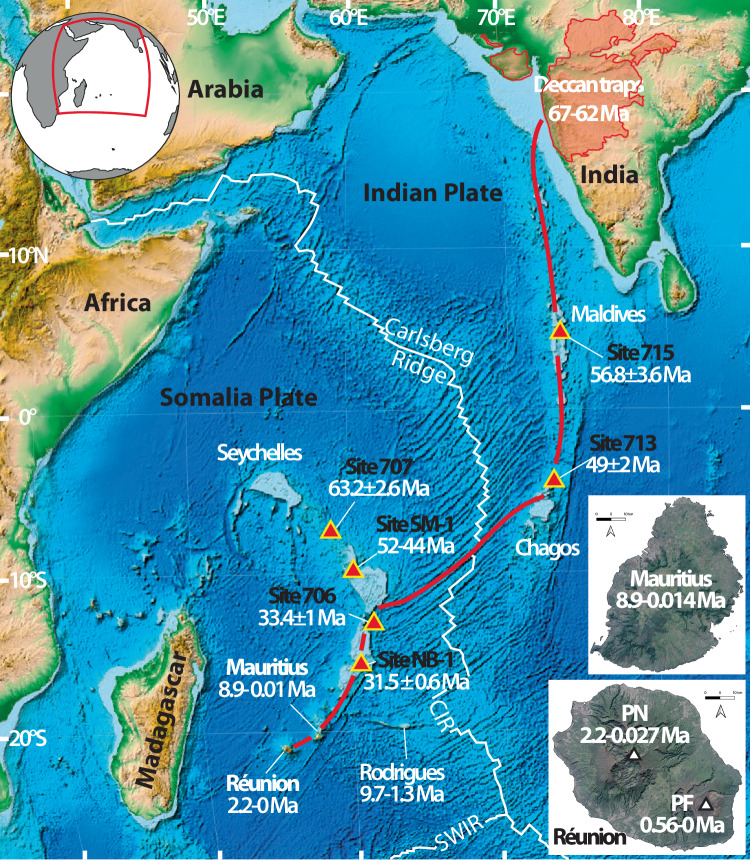
Topographic and bathymetric map of the western Indian Ocean. The map shows the presumed track of the Réunion hotspot (red line) from the Deccan Traps to Mauritius and Réunion. Rodrigues, the third island of the Mascarene Archipelago, off the hotspot track, is interpreted as caused by plume-ridge interaction^40^. Red and yellow triangles are drilling sites that recovered dated basaltic basement rocks^18^. The map uses GEBCO data^66^; insets are generated using Google Earth satellite images draped on digital elevation models (DEMs)^43,44^. PN, Piton des Neiges; PF, Piton de la Fournaise; CIR, Central Indian Ridge; SWIR, Southwest Indian Ridge. Maps data: GEBCO^66^ (main panel); Google © 2025 Airbus, SIO, NOAA, U.S. Navy, NGA, GEBCO (insets).

The Réunion hotspot is an emblematic setting to search for sub-Myr fluctuations in hotspot volcanism. Its surface expression began with the Deccan Traps at ~67 Ma (Fig. 1), which, together with the Chicxulub impact, is widely considered a major contributor to environmental disruption at the Cretaceous–Paleogene transition^15,16^. High-precision geochronology indicates that Deccan volcanism occurred in four discrete pulses separated by 100–400 kyr intervals^17^. The Réunion plume subsequently produced a chain of volcanic edifices on the Indian and Somali plates^18^ (Fig. 1). The question arises as to whether a sub-Myr variability is expressed in the recent Réunion hotspot as in the Deccan Traps. Seismic tomography suggests that the plume beneath the Réunion hotspot rises as a network of branches^19^ or ponding zones^20^ from the South-African Large Low Shear-Velocity Province at the core-mantle boundary. In the asthenosphere, the plume forms a broad low-shear-velocity zone hundreds of kilometer wide and inclined toward the Central Indian Ridge^21,22^. However, the spatial resolution of current tomography does not allow robust inferences on finer-scale structure within the plume conduit.

Here we investigate the temporal organization of volcanism in the two youngest volcanic edifices of the Réunion hotspot, Mauritius ( ≥ 8.9–0.014 Ma) and Réunion ( ≥ 2.2–0 Ma) islands (Fig. 1). Our approach differs from most previous studies in that it does not aim to infer magma production rates from erupted and intruded volumes, which typically yields a temporal resolution of ~1 Myr^2–4,14^. Instead, we combine field mapping, radiometric dating, and geomorphological constraints to reconstruct chronostratigraphies of volcanic activity and repose over the past 3 Ma, at a temporal resolution of ~10 kyr. We find that volcanic phases on Réunion and Mauritius are broadly coeval and recur on a few-hundred-kyr timescale, implying short-period modulation of magma supply within the Réunion hotspot.

## Results

### Former chronostratigraphies of the Mascarene Islands

A particularity of the Réunion hotspot is the unusually dense set of geochronological constraints on the two youngest islands of the Mascarene Archipelago, with >300 published radiometric ages from Réunion and >130 from Mauritius. This record documents construction and erosion on both islands at high temporal resolution. Réunion is composed of two volcanoes, the dormant Piton des Neiges (PN, Fig. 1) and the active Piton de la Fournaise (PF). The chronostratigraphy of Réunion has been updated repeatedly since the first geological map^23^, most recently through a synthesis combining geomorphological criteria and radiometric ages^24,25^. In this chronostratigraphy, Piton des Neiges is interpreted to have formed through three “shield building” phases of mafic volcanism separated by quiescence and erosion: La Montagne (LM; 2200–1800 ka), PN1 (1400–950 ka), and PN2 (600–430 ka). Piton des Neiges then emitted differentiated magmas during two “post-shield” phases, PN3 (340–180 ka) and PN4 (140–27 ka). The younger history of Piton de la Fournaise, still in a shield building stage, is subdivided into three or four periods of activity^25,26^, PF1 (560–290 ka), PF2 (250–60 ka), and PF3-4 (40–0 ka, with a caldera event between PF3 and PF4). Since publication of this chronostratigraphic chart, geomorphic and geochronological constraints of Réunion have improved substantially^27–32^, motivating a revision of the history of both volcanoes.

The chronostratigraphy of Mauritius Island, established in the 1970s^33,34^ and subsequently updated^35–38^, includes three phases of eruptive activity separated by quiescence and erosion: the Older Series ( > 8900–4700 ka), corresponding to the shield building stage; and the Intermediate Series (3500–1900 ka) and the Younger Series (1000–14 ka), interpreted as two phases of “rejuvenated” (i.e., away from the plume apex) mafic volcanism^39^.

Rodrigues, the third island of the Mascarenes and located off the presumed track of the Réunion hotspot (Fig. 1), is interpreted as the result of plume interaction with the Central Indian Ridge^40^. Compared with Mauritius and Réunion, Rodrigues has almost no geochronological information. Although a stratigraphy has been proposed for Rodrigues^41^, it is constrained by only two whole-rock K-Ar ages (1600–1300 ka) published before the development of modern dating protocols^42^.

Given the wealth of topographic, geological, and geochronological information now available for both islands, a detailed history of volcanism can be reconstructed. We combined digital elevation models (DEM; 5 m resolution for Réunion^43^, 30 m resolution Shuttle Radar Topography Mission–SRTM–for Mauritius^44^) with geological observations, mapping, literature radiometric ages, and ten additional radiometric ages by the unspiked Cassignol-Gillot K-Ar technique strategically selected to constrain key stratigraphic boundaries (see Methods). The full dataset of GIS layers and data Tables is accessible in open access^45^. The complete description of samples, protocols, and dating results is given in the Supplementary Material (Fig. S1, Supplementary Data 1). A filtering procedure (Methods) was applied to literature ages to enable comparison across different radiometric techniques and to retain the most reliable data for interpretation (Supplementary Data 2). Geological maps are provided in the Supplementary Material (Figs. S2, S3). The observations leading to our updated chronostratigraphies are summarized below.

#### Réunion Island

**Fig. 2:**
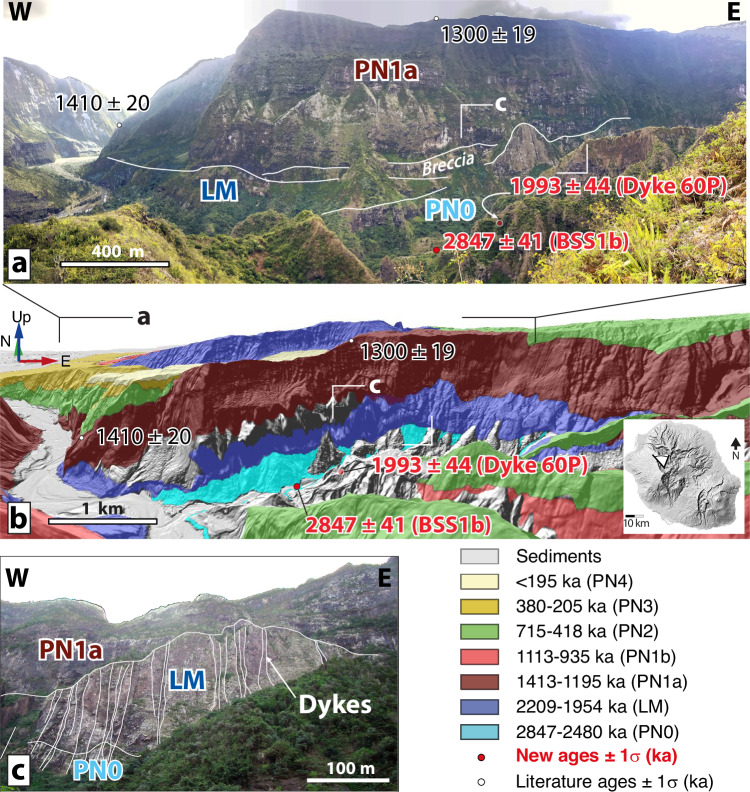
Construction and erosion pattern of the Bras de Sainte-Suzanne valley (Réunion Island). PN; Piton des Neiges; LM, La Montagne. The cliff is subdivided into lava-flow units (PN0, LM, and PN1a) separated by angular unconformities and volcaniclastic sedimentary breccias. **a** Outcrop view. **b** 3D view (5 m DEM^43^, no vertical exaggeration). **c** Detail of the cliff. The PN0 and LM units are cut by dykes (e.g., dyke 60P sampled for dating), whereas the PN1a unit is not. Scale bars apply to the foreground; scale varies with perspective.

A key outcrop for reconstructing the early subaerial history of Réunion is the northern cliff of the Sainte-Suzanne valley in the cirque of Mafate (Piton des Neiges volcano; Fig. 2 and S4a). This cliff exposes at least three lava flow units separated by two unconformities. Our age of 2847 ± 41 ka at the base of the cliff (sample BSS1b; Fig. 2a, b; all ages reported at 1σ) extends the geochronological record of Réunion by more than 600 kyr and provides strong evidence that the island was already emergent and at least ~300 m high at that time, well before the ~2200 ka initiation of the LM stage in previous chronostratigraphies^24,25^. This lowermost subaerial unit, which we term PN0, is eroded and unconformably overlain by an intermediate lava unit (Fig. 2a, b). Both the basal and intermediate units are cut by dykes from the northern rift zone of Piton des Neiges^46^ (Fig. 2c). We dated one such dyke at 1993 ± 44 ka (sample 60P; Fig. 2a, b). The intermediate unit must predate this dyke, and is attributed to the LM stage, whose lavas crop out nearby (Fig. 2a, b and S4a). The LM unit and dykes form an eroded relief that is unconformably overlain by a third unit (Fig. 2c). The absence of dykes in this third unit implies emplacement after cessation of the northern rift-zone activity and after 1993 ± 44 ka. Two literature ages bracket the base and top of this third unit at 1410 ± 20 ka and 1300 ± 19 ka, respectively (samples 99AF and 99AH in ref. ^24^), supporting attribution to the PN1 stage (Fig. 2a, b and S4a).

**Fig. 3:**
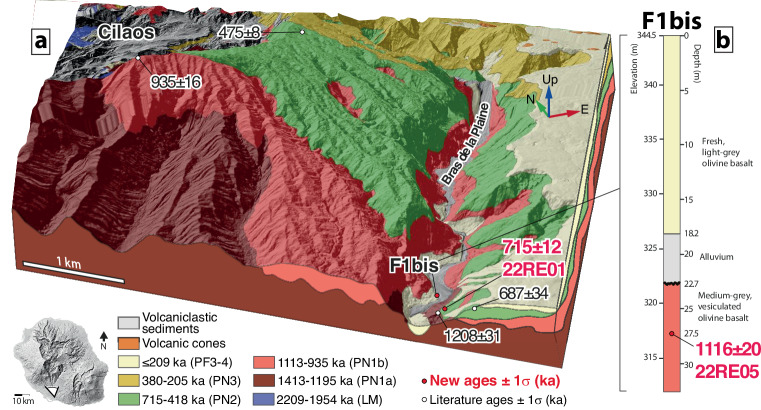
Construction and erosion pattern of the Bras de la Plaine valley (Réunion Island). **a** 3D view (5 m DEM^43^, no vertical exaggeration) and geological cross-section. Scale bar applies to the foreground; scale varies with perspective. **b** Log of the drill core “F1bis”, from which sample 22RE05 was collected (Lat. −21.2308°; Lon. 55.4977°). PN, Piton des Neiges; LM, La Montagne; PF, Piton de la Fournaise. Volcanic cones, overlying the other constructional units, are attributed to the PF3-4 period.

Another important area for the chronostratigraphy of Réunion is the southern flank of Piton des Neiges, which displays contrasted erosion patterns through time (Fig. 3 and S4b). A lower, deeply eroded series of mafic lava flows is dated by four ages ranging from 1247 ± 21 ka to 1187 ± 20 ka and falls within the PN1 stage (Fig. S4b; samples 99J in ref. ^24^, RU-63 and -64 in ref. ^47^, and 69H4 in ref. ^48^). An intermediate, moderately eroded series of mafic lavas overlies the lower series, producing a distinct topographic expression (Fig. 3a). We dated the base of the intermediate series at 1116 ± 20 ka (sample 22RE05; Fig. 3b), and its top is constrained by a literature age at 935 ± 16 ka (Fig. 3a and S4b; sample 99AY2 in ref. ^24^). Importantly, our sample occupies a lower structural position than an older sample from the Bras de la Plaine valley (sample 69H4 at 1208 ± 31 ka, ref. ^48^). This relationship implies an erosion interval between the two ages, and therefore requires subdivision of the PN1 stage into two phases separated by erosion, termed PN1a and PN1b. The southern flank of Piton des Neiges is unconformably covered by a third series of mafic lava flows that is only slightly eroded and forms a distinct, smoother slope morphology (Fig. 3a and S4b). Our age of 715 ± 12 ka (sample 22RE01) constrains the base of this series, whereas its top is dated at 475 ± 8 ka (sample 99BA in ref. ^24^), corresponding to the PN2 stage (Fig. 3a).

**Fig. 4:**
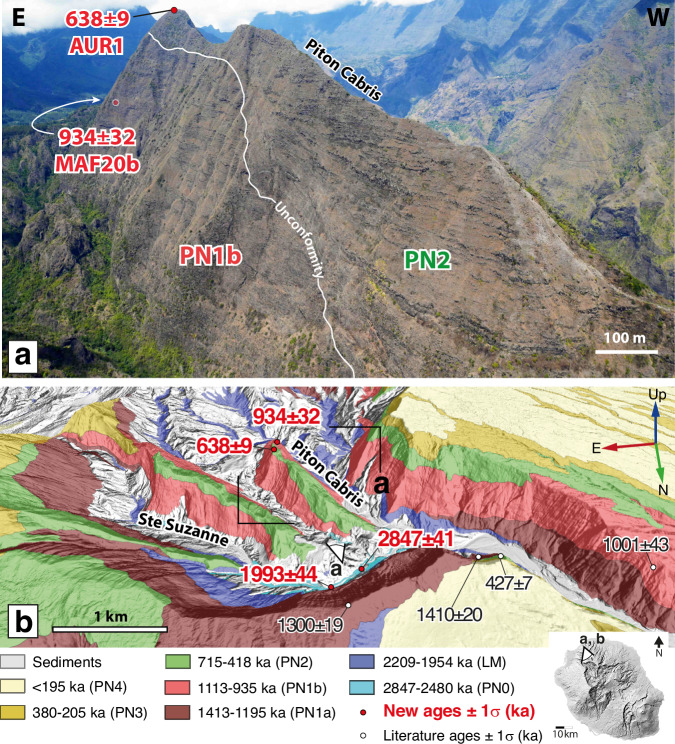
Piton Cabris ridge in the northern cirque of Mafate (Réunion Island). **a** Landscape view. **b** 3D view (5 m DEM^43^, no vertical exaggeration). Note the angular unconformity and age difference between lava-flow units, indicating inverted topography. PN and LM stand for Piton des Neiges and La Montagne, respectively. Scale bars apply to the foreground; scale varies with perspective.

Several lines of evidence allow us to generalize these inferences to the scale of Piton des Neiges (Supplementary Data 3). The Piton Cabris ridge in the cirque of Mafate provides one example (Fig. 4 and S4a). The ridge exposes a kilometer-long angular unconformity visible in the cliff (Fig. 4a). Below the unconformity, our age of 934 ± 32 ka (sample MAF20b; Fig. 4a), obtained at lower elevation than lava units dated 1410 ± 20 ka and 1300 ± 19 ka in the surrounding cliffs (Fig. 4b; S4a; samples 99AF and 99AH in ref. ^24^), indicates an inverted topography, i.e., lava flows emplaced in former topographic lows that are later preserved as topographic highs due to preferential erosion of older surrounding units. This geometry implies a phase of incision and erosion between the cliffs surrounding Mafate and the bottom of the cirque, followed by a partial refilling of the incised topography by lava flows. In addition, our age at 638 ± 9 ka (sample AUR1), above the unconformity of the Piton Cabris ridge (Fig. 4a), further requires an erosion interval between the PN1 and PN2 stages. Together, these two ages confirm the need to subdivide PN1 into two phases (PN1a and PN1b), followed by PN2, at the scale of the entire Piton des Neiges volcano. The PN2 stage is itself unconformably overlain by a unit dominated by plagioclase-rich lavas (PN3) dated from 380 ± 8 ka (samples 39T, 60a, and 48L2, ref. ^48^) to 205 ± 6 ka (samples 23-4 in ref. ^32^, and TK-4217 and 48L in ref. ^48^), producing an erosion contrast on the slopes of Piton des Neiges (e.g., Figs. 2, 3 and S2, S4). The PN3 unit is interrupted by a caldera event at ~200 ka^32^ followed by erosion and ignimbrite emplacement filling valleys. Differentiated volcanic activity resumes during PN4 from 195 ± 3 ka (sample 99O, ref. ^24^) to 26.3 ± 0.5 ka (RU-167, refs. ^31,49^).

In summary, the combination of geological mapping, radiometric ages, and geomorphological analysis leads to the following revised chronostratigraphy of Piton des Neiges (Supplementary Data 3): PN0 (2847–2480 ka), LM (2209–1954 ka), PN1a (1413–1195 ka), PN1b (1113–935 ka), PN2 (715–418 ka), PN3 (380–205 ka), and PN4 (195–27 ka).

Applying the same methodology to Piton de la Fournaise leads us to revise its chronostratigraphy as well. For instance, there is a clear geomorphic contrast between the eroded reliefs of the Plaine des Palmistes valley (dated from 561 to 399 ka, samples 57V and 57W in ref. ^48^; Fig. S5a) and the less eroded southwest slopes of Piton de la Fournaise (dated from 367 ka to 290 ka, samples RU76^47^ and 39D^50^). Moreover, an angular unconformity in the eastern cliff of the Rivière des Remparts valley suggests an erosion interval between these two units, consistent with a gap in radiometric data between 399 and 367 ka (Fig. S5b). Finally, ages from the base of the Rivière des Remparts valley (219 ± 2 ka or 180 ± 6 ka, samples 39V2^50^ and RU88^47^, respectively) are younger than the top of the cliff (291 ± 3 ka; sample 39D^50^), indicating an inverted topography and thus another erosion interval (Fig. S5b). We therefore revise the activity periods of Piton de la Fournaise as follows (Supplementary Data 3): PF1 (561–399 ka), PF2 (367– < 291 ka), and PF3-4 (209–0 ka).

#### Mauritius Island

**Fig. 5:**
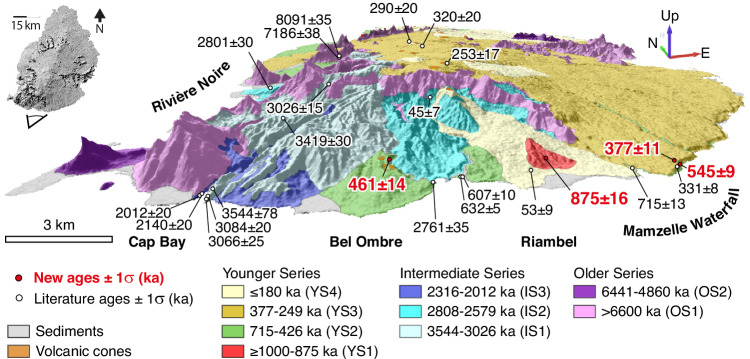
3D view of southern Mauritius. Our revised subdivision of stratigraphic units based on field mapping, radiometric ages (in ka), and geomorphology, is draped on the SRTM DEM^44^ (30 m resolution, x2 vertical exaggeration). OS, Older Series; IS, Intermediate Series; YS, Younger Series. Scale bar applies to the foreground; scale varies with perspective.

The stratigraphy of the Intermediate and Younger series of Mauritius also requires revision. In southern Mauritius, for example, the Rivière Noire valley is an erosion scar incising the Intermediate Series (IS; Fig. 5). The top of the incised surface is dated at 3026 ± 30 ka (sample MU85; ref. ^33^), whereas the valley is filled by lavas at 2808 ± 30 ka and 2801 ± 30 ka (samples MU49-50). The Rivière Noire valley was thus incised between ~3000 ka and ~2800 ka, suggesting a local interruption of volcanism within IS. Another example occurs at Cap Bay (southern Mauritius; Fig. 5 and S6). The eastern side of the bay forms a cliff, with average ages at its base and top of 3544 ± 156 ka (samples MU65 and 69; ref. ^33^) and 3066 ± 50 ka (MU70-72), respectively. In contrast, the western side is a gentle slope, with ages between 2140 ± 40 ka (samples MU62-63; ref. ^33^) and 2012 ± 40 ka (MU61). This age difference and the contrasted morphologies imply a second case of erosion and valley infill between ~3000 ka and ~2200 ka within IS. In the central north of Mauritius, borehole stratigraphic correlations reveal at least two unconformities between two drill core samples dated at 2579 ± 30 ka and 2104 ± 10 ka (samples B18-2 and -5; ref. ^37^), providing a third example of erosion within the IS unit (Fig. S7). The systematic occurrence of erosion intervals wherever the IS is observed leads to subdivide this unit into three phases separated by volcanic quiescence and erosion (Fig. 5; Supplementary Data 3): IS1 (3544–3026 ka), IS2 (2808–2579 ka), and IS3 (2316–2012 ka).

Multiple lines of evidence show that the Younger Series must also be subdivided into shorter periods of magmatic activity and repose. At Riambel (Southern Mauritius; Fig. 5 and S8) an eroded surface, which we dated at 875 ± 16 ka (sample 19MU12; Figure S8), is overlain by lava flows dated from 715–607 ka (sample MU127 and 56; ref. ^33^) to 53–45 ka (samples 19MU14 and 07; ref. ^38^). This age confirms that activity ended at ~875 ka and indicates subsequent repose and erosion, followed by renewed volcanism at ~715 ka in southern Mauritius. The Mamzelle Waterfall provides another example of a volcanic hiatus between two lava flow units (Fig. 5 and S9). Altered picritic lavas at the base of the waterfall are overlain by a palaeosol and yield a K-Ar age of 545 ± 9 ka (19MU05; Fig. S9). A fresh, prismatic basalt flow forming the waterfall and unconformably overlying the palaeosol yields a K-Ar age of 377 ± 11 ka (19MU06; Fig. S9), indicating a hiatus marked by soil development between these ages. Similar relationships occur elsewhere, for example in the Rivière de l’Est valley (eastern Mauritius; Fig. S10), where an unconformity is bracketed by ages of 426 ± 35 ka (sample MU101; ref. ^33^) and 113 ± 9 ka (sample 19MU09; ref. ^38^). These unconformities suggest a period of island-wide quiescence and erosion between ~426 ka and ~377 ka, consistent with a gap in ages across this interval. Likewise, geomorphological criteria (slope changes, erosion patterns) and a gap in ages suggest that volcanic activity halted or at least slowed down between ~249 ka and ~180 ka (Fig. 5 and S11). In summary, our observations lead us to refine the Younger Series (YS) into four phases (Supplementary Data 3): YS1 ( ≥ 1000–875 ka), YS2 (715–426 ka), YS3 (377–249 ka), and YS4 (180–14 ka).

## Discussion

### Synchronized volcanism over the Mascarene Islands

**Fig. 6:**
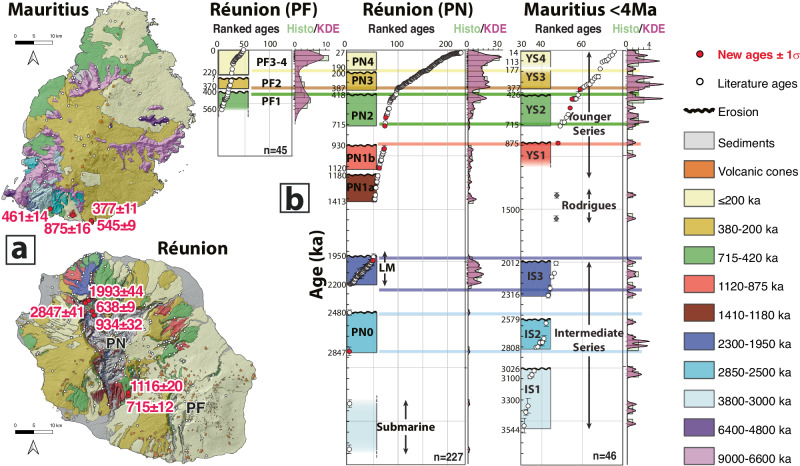
Revised geological history of Réunion and Mauritius. **a** Geological maps draped on the Réunion and Mauritius DEMs^43,44^. **b** Chronostratigraphic charts based on our subdivision of volcanic slopes into lava-flow units (Supplementary Data 3). PN, Piton des Neiges; PF, Piton de la Fournaise; IS, Intermediate Series; YS, Younger Series. Histo and KDE denote histogram and kernel density estimate plots, respectively, both generated using IsoplotR. Error bars on ages are displayed at the 1 SD level.

When placed side by side, the revised maps and chronostratigraphies of Réunion and Mauritius islands reveal a striking level of correspondence over the past ~3 Ma (Fig. 6a, b; Supplementary Data 3). For instance, the PN0 period in Réunion (2847 ± 40 – 2480 ± 25 ka) nearly coincides within 1σ uncertainty with the IS2 period in Mauritius (2808 ± 15 – 2579 ± 30 ka), and fully coincides at 2σ (Fig. 6b and S12). Likewise, the LM period of activity in Réunion (2209 ± 7 – 1954 ± 30 ka) nearly overlaps within 1σ uncertainty with the IS3 period in Mauritius (2316 ± 25 – 2012 ± 20 ka), and both phases are followed by erosion intervals. The PN1a stage in Réunion (1413 ± 21 – 1187 ± 20 ka) represents an exception to this synchronicity, with no clear equivalent in Mauritius. We note, however, that Rodrigues was active during this interval, suggesting possible coeval activity elsewhere in the Mascarene Islands (Fig. 6b; Supplementary Data 3), although geochronological constraints remain too sparse to assess its volcanic tempo in detail.

Subsequently, the PN1b period ends with an unconformity and erosion event (934 ± 32 ka), contemporaneous with the termination of the YS1 period (875 ± 16 ka; Fig. 6b). The correspondence is even clearer for PN2 (715 ± 12 – 418 ± 8 ka), PF1 ( ≥ 561 ± 5 – 399 ± 4 ka), and YS2 (715 ± 13 – 426 ± 35 ka), all interrupted by erosion (Fig. 6b and S12). After this repose, volcanic activity resumes nearly synchronously at the three eruptive centers during PN3 (387 ± 9 – 207 ± 11 ka), PF2 (367 ± 9 – ≤291 ± 3 ka), and YS3 (377 ± 11 – 249 ± 22 ka). The most recent history likewise shows broadly coeval activity of PN4 (195 ± 3 – 27 ± 1 ka), PF3-4 (209 ± 10 – 0 ka), and YS4 (177 ± 7 – 14 ± 4 ka).

There is thus a synchronized rhythmicity of volcanic phases and repose intervals among two eruptive centers from ~3000 ka to ~560 ka, and among three centers thereafter (Fig. 6; Supplementary Data 3). Phases of volcanic activity lasted on average 221 ± 73 kyr over the past 3 Ma (minimum PF2, 117 kyr; maximum PN0, 367 kyr, Supplementary Data 3), with an average recurrence interval of 370 ± 202 kyr between successive phases (minimum 149 kyr from PF1 to PF2; maximum 778 kyr from LM to PN1a).

As with any stratigraphic reconstruction, our subdivision into volcanic phases is inherently constrained by exposure and preservation, and constructional and erosional processes may operate simultaneously in different sectors of shield volcanoes. The phases defined here, therefore, represent intervals of regionally enhanced or diminished volcanic output rather than strictly synchronous volcano-wide on/off behavior. Moreover, the resolution of our chronostratigraphies decreases backward in time, as older volcanic units are progressively removed by erosion, buried, or less densely sampled. The apparent shortening of repose intervals toward the present, particularly over the last million years, may thus partly reflect improved preservation and a higher density of radiometric ages in younger successions (Fig. 6b). Importantly, the ~400 kyr modulation of magmatic fluxes inferred in this study does not rely on a single section, but emerges from the repeated correspondence of erosion surfaces and radiometric age clusters across multiple, spatially independent sites on three eruptive centers and two separate islands. This redundancy supports the existence of a coherent signal beyond local geomorphic variability. We therefore interpret the synchronized chronostratigraphies of Réunion and Mauritius as reflecting fluctuations in magma supply rates through time. Although volumetric reconstruction would require full 3D modeling beyond the scope of this study, the duration of constructional intervals implies sustained variations in magma flux rather than isolated eruptive events. These fluctuations appear independent of chemical and isotopic variations among the three eruptive centers (Fig. S13; Supplementary Data 4, 5), suggesting a common synchronization mechanism despite different melting sources and ascent pathways.

### Melting pulsation of the Réunion plume?

Which mechanism is responsible for the synchronization of volcanism in the Réunion hotspot across two islands over at least 3 Ma? The wavelength of synchronized volcanism ( ≥ 230 km) exceeds the thickness of the oceanic lithosphere ( ≤ 70 km). This lithosphere is heterogeneous in thickness ( ~70 km beneath Réunion and ≤50 km beneath Mauritius), structure (Moho at ~12 km beneath Réunion and 17–28 km beneath Mauritius), and amount of magmatic underplating ( ~2 km beneath Réunion and ~7 km beneath Mauritius)^51^. Synchronization of volcanic activity across lithospheric domains of contrasting thickness and structure, therefore, argues against shallow-level storage-controlled cyclicity alone. Independent shallow magma storage systems separated by ~230 km would be unlikely to exhibit sustained, temporally coherent eruptive behavior over several million years without a common mantle-scale driver. The observed rhythmicity is thus most consistent with a process operating deeper than the lithosphere, reflecting quasi-periodic modulation of plume-related melt generation and/or mantle-controlled melt migration.

Based on Nd-Pb-Hf isotopic variations, Bosch et al. ^52^ proposed that magmas from Réunion Island sampled different “blobs” of the mantle plume through time. A re-examination of Sr-Nd-Pb isotopic data (Supplementary Data 5) shows that Mauritius and Réunion have distinct isotopic compositions despite coeval periods of magmatic activity (Fig. S13). In particular, Piton des Neiges and Piton de la Fournaise display more radiogenic signatures than the Intermediate and Younger series of Mauritius, suggesting a larger contribution of plume-derived material in Réunion magmas and a greater contribution of MORB-like mantle in Mauritius^53^.

**Fig. 7:**
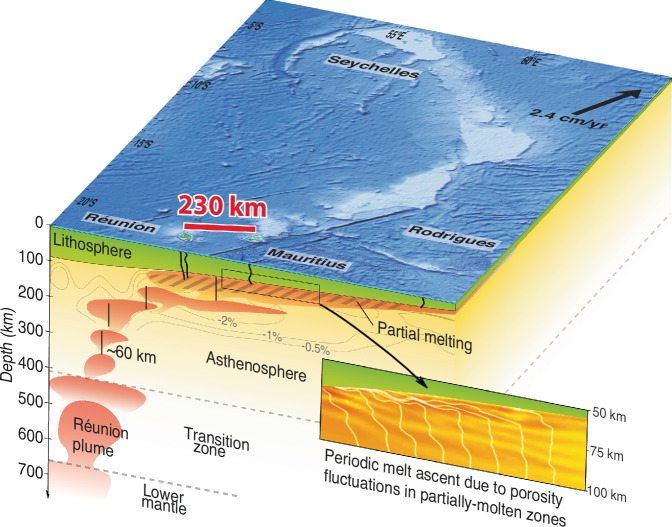
Conceptual model of Réunion plume pulsation. The model incorporates the synchronized volcanism of Réunion and Mauritius, geochemical heterogeneities^37,52,53^, plume deflection toward the Central Indian Ridge (with isocontours of isotropic shear-wave velocity anomalies, in percent^22^), and lithosphere thicknesses^51^. The plume periodically conveys quanta of rising hot material beneath the oceanic lithosphere, due to deflection in the asthenosphere^10^ or interactions with the transition zone^11,12^. Each quantum spreads beneath the lithosphere-asthenosphere boundary as a “pancake” and delivers coeval melt supply to eruptive centers separated by ~230 km. The ~400 kyr pulsation of the Réunion hotspot may arise from plume quanta separated by ~60 km (vertical black bars) in the asthenosphere (assuming a plume ascent rate of 15 cm/yr^12^), and/or from porosity fluctuations in the partially molten zone leading to periodic magma ascent rates^55,56^. Map data: Google © 2025, SIO, NOAA, U.S. Navy, NGA, GEBCO, Image Landsat / Copernicus.

However, volcanic synchronization challenges the concept of independent “blobs” feeding Réunion and Mauritius separately, and instead suggests that the melting region is sufficiently extensive to supply Piton des Neiges, Piton de la Fournaise, and Mauritius simultaneously, while remaining compositionally heterogeneous. Synchronized volcanism, compositional variability, and geophysical observations may be reconciled in a scenario where rising quanta of Réunion plume material spread at the base of the lithosphere as discrete “pancakes”^54^, leading to a plume pattern inclined toward the Central Indian Ridge^22^ (Fig. 7). In this framework, each plume pancake initiates heterogenous partial melting, likely within the asthenospheric low-velocity zone at 80–120 km depth identified by seismic receiver functions^51^. These melts, generated at comparable depths beneath Réunion and Mauritius, may ascend at similar rates, facilitating broadly coeval magmatic activity on both islands. As previously noted^51,52^, Réunion magmas record a greater contribution of plume material because the island is presently located closer to the apex of the rising plume (Fig. 7 and S13).

An important question is whether the ~400 kyr rhythmicity recorded by the Mascarene chronostratigraphies reflects periodic melt generation within the Réunion plume, cyclic melt extraction and transport from a more continuously melting source, or a combination of both processes. Most mechanisms proposed to explain plume pulsations in hotspots and large igneous provinces (e.g., viscosity and/or density contrasts between plume and ambient mantle, instabilities in tilted conduits, or processes involving the transition zone) predict periodicities of 1–20 Myr for realistic parameter values^5–8,10–13^. The rhythmicity documented here, approximately one order of magnitude shorter than these predictions, would therefore require either extreme parameter values in such models or additional mechanisms. Assuming reasonable plume ascent rates of ~15 cm/yr^12,55^, successive plume pulses would need to be vertically separated by ≤60 km within the asthenosphere to generate melt production pulses at ~400 kyr intervals (Fig. 7). Current seismic tomography models^21,22^ lack sufficient vertical resolution to directly test this possibility.

Alternatively, the short-period volcanic signal in the Mascarene Islands may arise from cyclic melt transport beneath the lithosphere driven by “porosity waves”, i.e., fluctuations of mantle porosity and permeability induced by rising melts (Fig. 7). Two-phase flow simulations beneath mid-ocean ridges have demonstrated the propagation of porosity waves below the lithosphere, capable of generating pulsing melt migration with periodicities of 100–500 kyr^56^. In a related framework, Ghosh et al. ^55^ proposed that plume pulses may trigger melting pulses at intervals of 100–400 kyr, as accumulated melts transiently enhance mantle rock permeability below the lithosphere-asthenosphere boundary, leading to oscillatory melt extraction and transport. Such melt-delivery pulses have been invoked to explain the Deccan Traps eruptive history between ~66.3 and ~65.5 Ma as a succession of four magmatic episodes separated by 150–400 kyr^17,55^. This periodicity is strongly reminiscent of the Mascarene magmatic tempo over the past 3 Ma. Such correspondence suggests that the ~400 kyr cyclicity of magmatism may represent a long-lived characteristic timescale of the Réunion hotspot system, from the Deccan Traps to the present-day Mascarene Islands. Numerical modeling that couples plume ascent with partial melting and melt transport will be required to test and quantify this interpretation.

Finally, our study raises the question of the uniqueness of the short-period rhythmicity documented for the Réunion hotspot. Similar sub-Myr variations in melt delivery may exist in other hotspots but remain unresolved owing to insufficient temporal resolution. Comparable 200–400 kyr-long phases of volcanic activity and repose have been reported in several intraplate islands of the Indian Ocean (Supplementary Data 6), although these have not been explicitly examined for periodic behavior. In well-studied systems such as Hawai'i, dense stratigraphic and geochronological datasets are available, yet sub-Myr cyclicity has not been systematically evaluated in terms of synchronization across multiple volcanoes. Our results, therefore, underscore the need for comparable high-resolution chronostratigraphic analyses in other hotspot systems before assessing the broader significance of the ~400 kyr cyclicity identified here for mantle-derived magmatism.

## Methods

Field surveys were conducted on Réunion and Mauritius to refine the subdivision of lava flows into stratigraphic units separated by hiatuses of deposition and/or erosion. Evidence for hiatuses includes angular unconformities, sedimentary breccia horizons or palaeosols between lava units, and geomorphic indicators of volcanic renewal following erosion, such as inverted topography and filled valleys, and contrast of erosion patterns on volcano slopes as observed in DEMs. Stratigraphic correlations are necessarily constrained by exposure and preservation of volcanic units; correlations were therefore based on the combination of independently observed unconformities across multiple sections and volcano slopes.

Samples were collected for K-Ar dating to place absolute constraints on the lower and upper bounds of stratigraphic units, or to assess the validity of radiometric ages available in the literature. In the field, we preferentially sampled massive rocks with the highest possible freshness and the lowest possible vesicularity, as these criteria are critical for successful K-Ar dating. Outer parts of samples in contact with seawater or meteoric water were removed to minimize incorporation of K from seawater and of potential excess radiogenic ^40^Ar from rapidly cooled rinds^57^.

The choice of the unspiked Cassignol-Gillot K-Ar technique was dictated by our objective of dating mafic rocks with low concentrations of radioactive and radiogenic elements over the subaerial history of Réunion and rejuvenated Mauritius ( ~3.5 Ma). Applied to carefully separated groundmass, this approach supersedes earlier whole-rock K-Ar dating by removing xenoliths and alteration phases that may bias results. The unspiked Cassignol-Gillot K-Ar technique also avoids the drawbacks associated with sample irradiation and its recoil effect, leading to the interfering production of ^36^Ar, which can compromise ^40^Ar/^39^Ar precision for young (< 1 Ma), low-K, and high-Ca rocks such as basalts^58^. It is also widely applicable to Quaternary basalts, whereas other techniques may be limited by the scarcity or absence of suitable accessory minerals (zircon, apatite, sanidine, etc.), or by low concentrations of radioactive and radiogenic isotopes.

We applied the unspiked Cassignol-Gillot K-Ar technique to carefully selected groundmass separates. Samples were manually crushed and sieved, then ultrasonically cleaned in 10% HNO_3_. Fractions were isolated within a narrow density interval by heavy liquid separation using diiodomethane to remove dense xenocrysts and the lightest phases in case of undetected alteration. Indeed, incorporation of xenocrysts or altered phases can yield K-Ar ages older than “true” cooling ages^38,59^. Potassium and argon measurements followed the unspiked Cassignol-Gillot method^58,60,61^ (see Supplementary Information), with duplicate measurements. Unless otherwise stated, uncertainties are reported at the 1σ level (Supplementary Data 1).

To interpret our K-Ar ages in the context of literature data (U-Pb, (U-Th)/He, ^40^Ar/^39^Ar, U-series disequilibrium, and ^14^C), we applied a filtering procedure adapted from Michon et al. ^62^ to build a harmonized database for the Mascarene Islands (Supplementary Data 2). This procedure includes (1) rejection of ^40^Ar/^39^Ar ages that do not meet standard quality criteria (plateau and isochron ages inconsistent); (2) rejection of K-Ar ages lacking uncertainties (e.g., ref. ^35^) or obtained from rocks described as altered (e.g., ref. ^63^); (3) rejection of U-series disequilibrium ages, which are systematically younger than ages obtained by other techniques for the same rocks^25,31,64^; (4) rejection of ages with large relative uncertainties using the following 1σ cut-offs: >7.5% for ages >5 Ma; >10% for 2.5–5 Ma; >15% for 1–2.5 Ma; >25% for 0.25–1 Ma; and >50% for <0.25 Ma; (5) recalculation of all K-Ar ages using the decay constants of Steiger and Jäger^65^; and (6) homogenization of all uncertainties to the 1σ level. Ages and durations are expressed in ka and kyr, respectively, to maintain consistent numerical precision and facilitate direct comparison of uncertainties across Quaternary and late Neogene timescales.

Finally, the radiometric age database was combined with the trace element and Sr-Nd-Pb-Hf isotopes database of Nauret et al. ^53^ to build geochemical and isotopic temporal series (Fig. S13 Supplementary Data 4, 5).

## Supplementary information

Supplementary Information

Description of Additional Supplementary Files

Supplementary Data 1

Supplementary Data 2

Supplementary Data 3

Supplementary Data 4

Supplementary Data 5

Supplementary Data 6

Transparent Peer Review file

## Data Availability

Sample descriptions, geological maps, filtering criteria, geochemical analyses, and analytical protocols are available in the Supplementary Data. Additional views of outcrops and landscapes are also provided in the Supplementary Information. The full dataset of GIS layers and supplementary Tables generated in this study are available in the Figshare database under accession code 10.6084/m9.figshare.29098955 (10.6084/m9.figshare.29098955.v2).
