# Supplementary material for: Synchronized Mascarene volcanism reveals 400 kyr cycles in melt supply from the Réunion plume
Source: Nat Commun. 2026 May 7;17:6118. doi: 10.1038/s41467-026-72855-1 (PMC13358134; doi:10.1038/s41467-026-72855-1)
Supplement: Supplementary file 1 — Supplementary Information [file 41467_2026_72855_MOESM1_ESM.pdf]

Supplementary Information
Synchronized Mascarene volcanism reveals 400 kyr cycles in melt supply from the Réunion plume

This Supplementary Information includes: (1) a description of the samples used for K-Ar dating; (2) a summary of the unspiked Cassignol-Gillot technique employed for K-Ar dating; (3) descriptions of the Supplementary Data cited in the main text; and (4) the Supplementary Figures supporting the main text.

1. Sample description

Réunion samples

BSS1b: Basaltic lava flow, taken in the dry riverbed of the Bras de Sainte-Suzanne River (north side of the cirque of Mafate, Piton des Neiges volcano), east and upstream of the Bras des Merles tributary. The lava flow covers a rubefacted level of altered volcanic breccia. Both the breccia and the lava flow are intruded by numerous subvertical dykes oriented N120 – 160°E. The basalt of sample BSS1 is fresh and slightly vesicular, with a light-grey matrix containing ~5 vol% olivine microphenocrysts (≤1 mm in diameter; Fig. S1).

60P: Basaltic dyke cutting basal units exposed in the Bras de Sainte-Suzanne River (north side of the cirque of Mafate, Piton des Neiges volcano), including the lava flow from which sample BSS1b was collected. This sample was originally collected by Kluska (1997)[1]. The basalt is massive and contains ~20 vol% olivine phenocrysts (≤5 mm in diameter) embedded in a dark-grey matrix (Fig. S1).

MAF20b: Basaltic lava flow collected within the southern cliff of the Piton Cabris ridge (north side of the cirque of Mafate, Piton des Neiges volcano), near Bord Bazar along the Grand Place trail, below the unconformity visible in the cliff. The basalt is massive and displays a dark blue-grey matrix containing ~5 vol% olivine microphenocrysts (≤1 mm in diameter; Fig. S1).

AUR1: Basaltic lava flow collected at the summit of the Piton Cabris ridge (north side of the cirque of Mafate, Piton des Neiges volcano), above the angular unconformity visible on the northern cliff. The basalt is slightly vesicular, with light-grey matrix and up to ~10 vol% olivine phenocrysts (≤5 mm in diameter; Fig. S1).

22RE05: Basaltic lava flow sampled from core 16 of the F1bis borehole, drilled in the riverbed of the Bras de la Plaine valley, at the junction of Piton des Neiges and Piton de la Fournaise volcanoes. The sample was collected at a depth of 27.5 m in the borehole (corresponding to 316.8 m above sea level). It consists of fresh, vesicular, medium-grey olivine basalt containing ~15 vol% olivine phenocrysts (≤5 mm in diameter; Fig. S1).

22RE01: Basaltic lava flow collected in the eastern cliff of the Bras de la Plaine valley, along the Pont d'Yves trail, at the junction of Piton des Neiges and Piton de la Fournaise volcanoes. The basalt is fresh, vesicular, with a light-grey matrix containing ~15 vol% olivine microphenocrysts (≤1 mm in diameter; Fig. S1). Olivine microphenocrists are slightly transformed into hiddingsite.

Mauritius samples

19MU12: Approximately 10 m^3 boulder of basalt unearthed during excavation works from a 1-m-thick lateritic soil on the slopes of the Chamouny locality, about 1 km west of Surinam, in the Riambel area (south coast of Mauritius). The basalt is fresh and massive, with a medium-grey matrix containing ~10 vol% olivine phenocrysts (≤5 mm in diameter; Fig. S1). The rock also contains pegmatoid cavities filled with acicular microphenocrists of olivine, clinopyroxene, and plagioclase.

19MU01: Basaltic boulder extracted from altered lava flows on the western slopes of the Jacotet river, Bel Ombre locality, southern Mauritius. The fresh core of the boulder contains massive basalt with a plagioclase-rich microlithic matrix (Fig. S1).

19MU05: Basaltic boulder extracted from altered lava flows exposed in the riverbed on the western side of the Mamzelle waterfall, east of the Gris Gris locality (southern Mauritius). The lava belongs to a series of flows covered by a ~3 m thick lateritic palaeosol, which is itself unconformably overlain by unaltered lava flows. The fresh core of the boulder consists of massive, dark-grey, aphyric basalt containing olivine and plagioclase microliths (Fig. S1).

19MU06: Columnar-jointed basaltic lava flow forming the Mamzelle waterfall, unconformably overlying the palaeosol and the lavas from which sample 19MU05 was collected. The sample was taken from the western side of the riverbed at the top of the waterfall. The basalt is fresh, massive, and aphyric, with a dark-grey matrix containing olivine and plagioclase microliths (Fig. S1).

2. Details of the Cassignol-Gillot technique for K-Ar dating

Potassium contents were measured by flame-absorption spectrometry using BCR-2 (K = 1.481 %; ref.[2]) and MDO-G (K = 3.51 %; ref.[3]) as reference standards. Argon, along with other gases, was extracted after complete melting of the sample at high temperature (>1400°C). A three-step purification procedure was then applied to remove all gases except noble gases (primarily Ar). First, gas clean-up was performed using 15 g of pure Ti foam heated at 800°C for one hour and subsequently cooled to room temperature for about 20 minutes. Two additional purification steps of 2 minutes were then conducted using Al-Zr AP10GP SAES getters to further purify gases prior to analysis. The ^{36}Ar and ^{40}Ar isotopes were measured using a multi-collector 180° sector mass spectrometer by comparing the samples and atmospheric aliquots[4]. The ^{40}Ar signal was calibrated by an air pipette compared to the HD-B1 standard[5,6] using the age of 24.18 Ma (ref.[7]). Calculations employed the ^{40}K decay constants and K isotopic ratio of [8].

3. Supplementary Data

The full set of Supplementary Tables used in this study is available in a Figshare repository (https://doi.org/10.6084/m9.figshare.29098955.v2) and as Supplementary Data.

Supplementary Data 1: K–Ar ages from this study. Column headings include sample names; latitude and longitude (decimal degrees); potassium (K) concentration (wt%); concentration of radiogenic argon ($^{40}Ar^*$; %); concentration of $^{40}Ar^* \times 10^{11}$ (atoms per gram); age (ka); 1σ uncertainty (ka); weighted mean age (ka); and 1σ weighted mean uncertainty (ka).

Supplementary Data 2: Compilation and filtering of literature radiometric ages for the Mascarene Islands. Data for Piton des Neiges volcano are from refs[1,9–27]. Data for Piton de la Fournaise are from refs[1,9,14,19,28–31]. Ages for Mauritius and Rodrigues are from refs[21,32–37].

Supplementary Data 3: Summary of geological observations and radiometric ages used to subdivide the history of the Mascarene Islands into chronostratigraphic units.

Supplementary Data 4: Compilation of trace element data combined with radiometric ages (Supplementary Data 2) from the Mascarene Islands. Data are from refs[9,14,16,18,32,34,36,38–44].

Supplementary Data 5: Compilation of Sr-Nd-Hf-Pb isotopic data combined with radiometric ages (Supplementary Data 2) from the Mascarene Islands. The isotopic database and filtering procedure follow Nauret at al.[41]. Data are from refs[9,31,32,36,38–60].

Supplementary Data 6: Compilation of chronostatigraphic charts for Marion and Prince Edward Islands, Possession and East Islands (Crozet Archipelago), the Kerguelen Archipelago, and Heard Island over the Upper Miocene and Quaternary. Sources are from refs[61–71].

4. Supplementary Figures

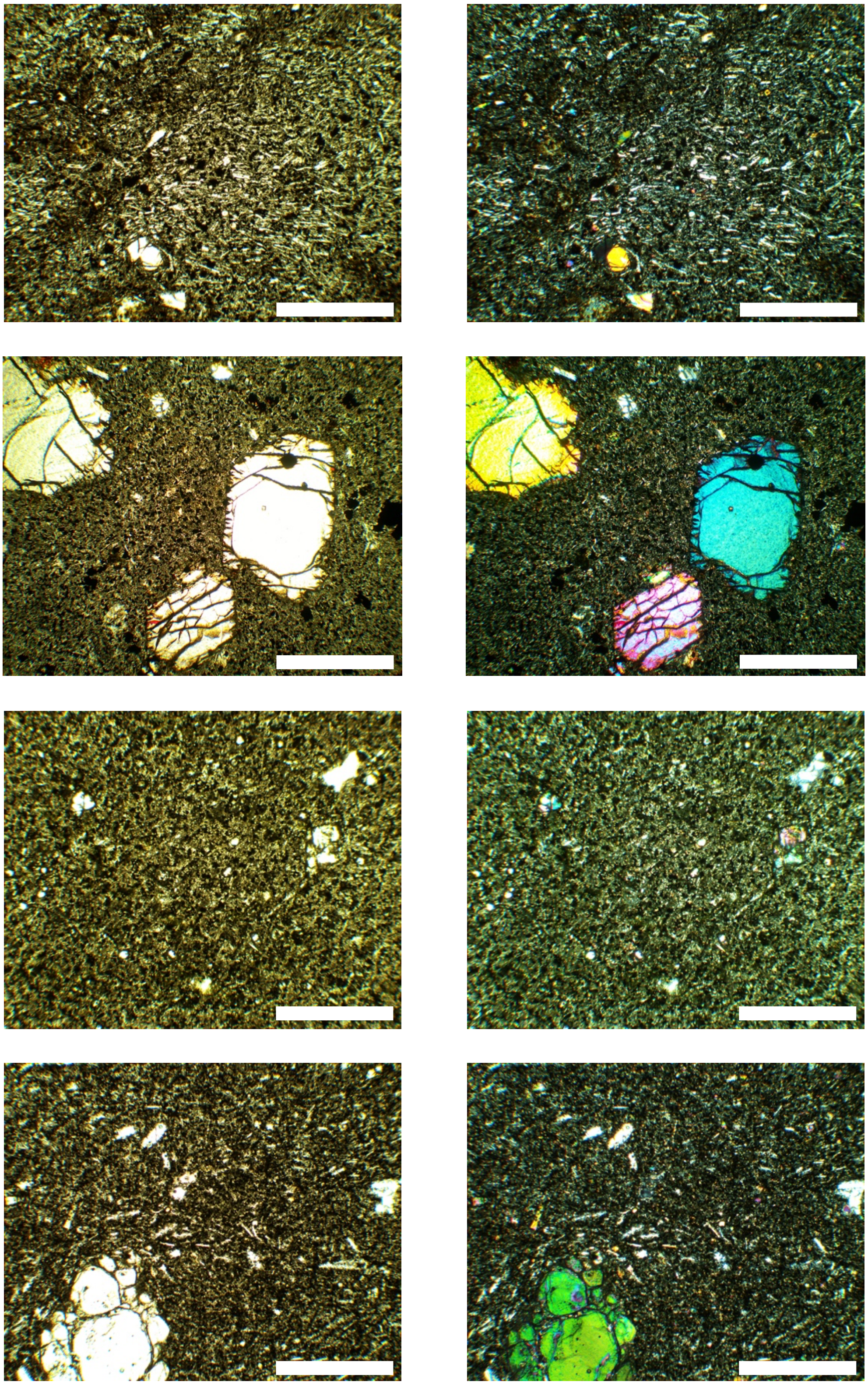

BSS1b

60P

MAF20b

AUR1

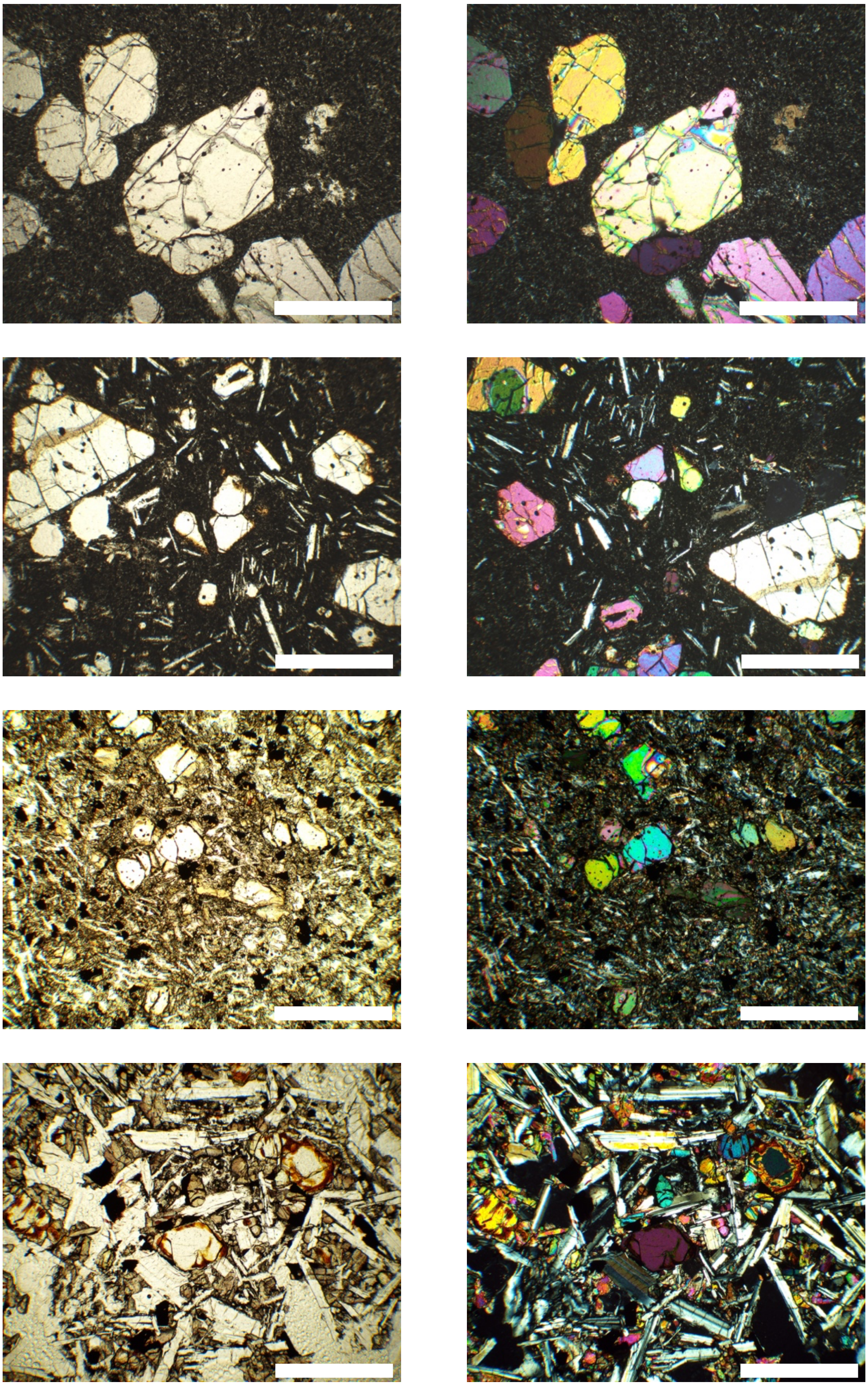

22RE05

22RE01

19MU12

19MU01

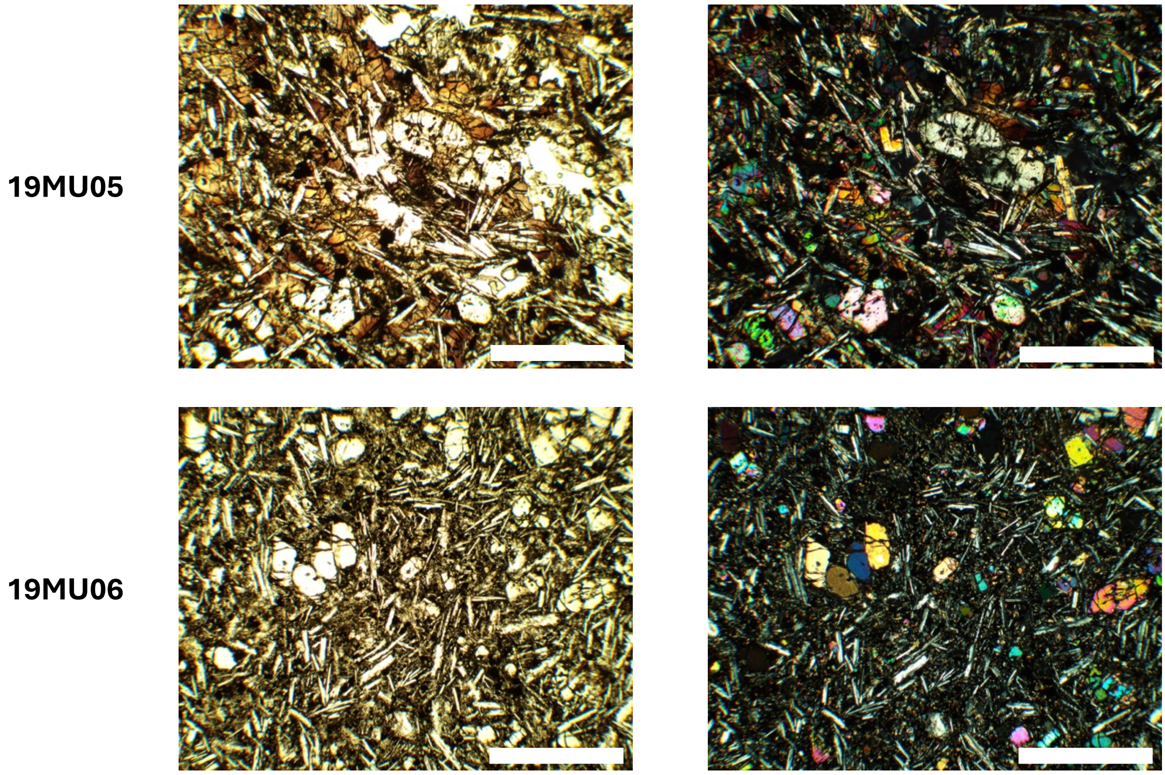

Figure S1: Thin section photomicrographs of samples dated using the unspiked Cassignol-Gillot K-Ar method on groundmass (see Supplementary Data 1 for analytical data). Left: plane-polarized light; right: cross-polarized light. All images were acquired at the same magnification (scale bar = 1 cm).

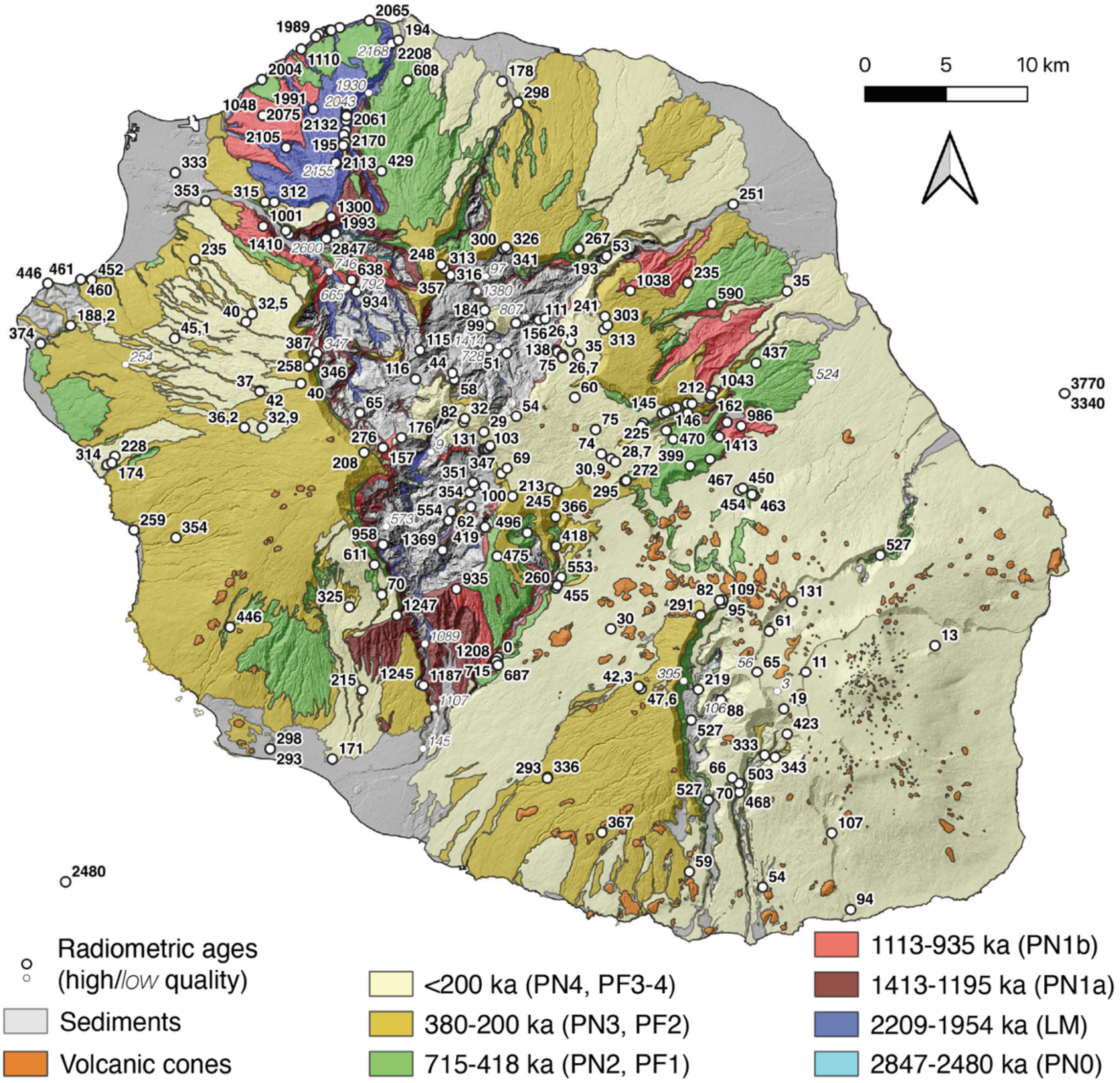

Figure S2: Geological map of Réunion Island based on field observations, radiometric ages, and geomorphological analysis. PN, Piton des Neiges; LM, La Montagne; PF, Piton de la Fournaise. Geology layers are draped on a 5-m-resolution digital elevation model[72].

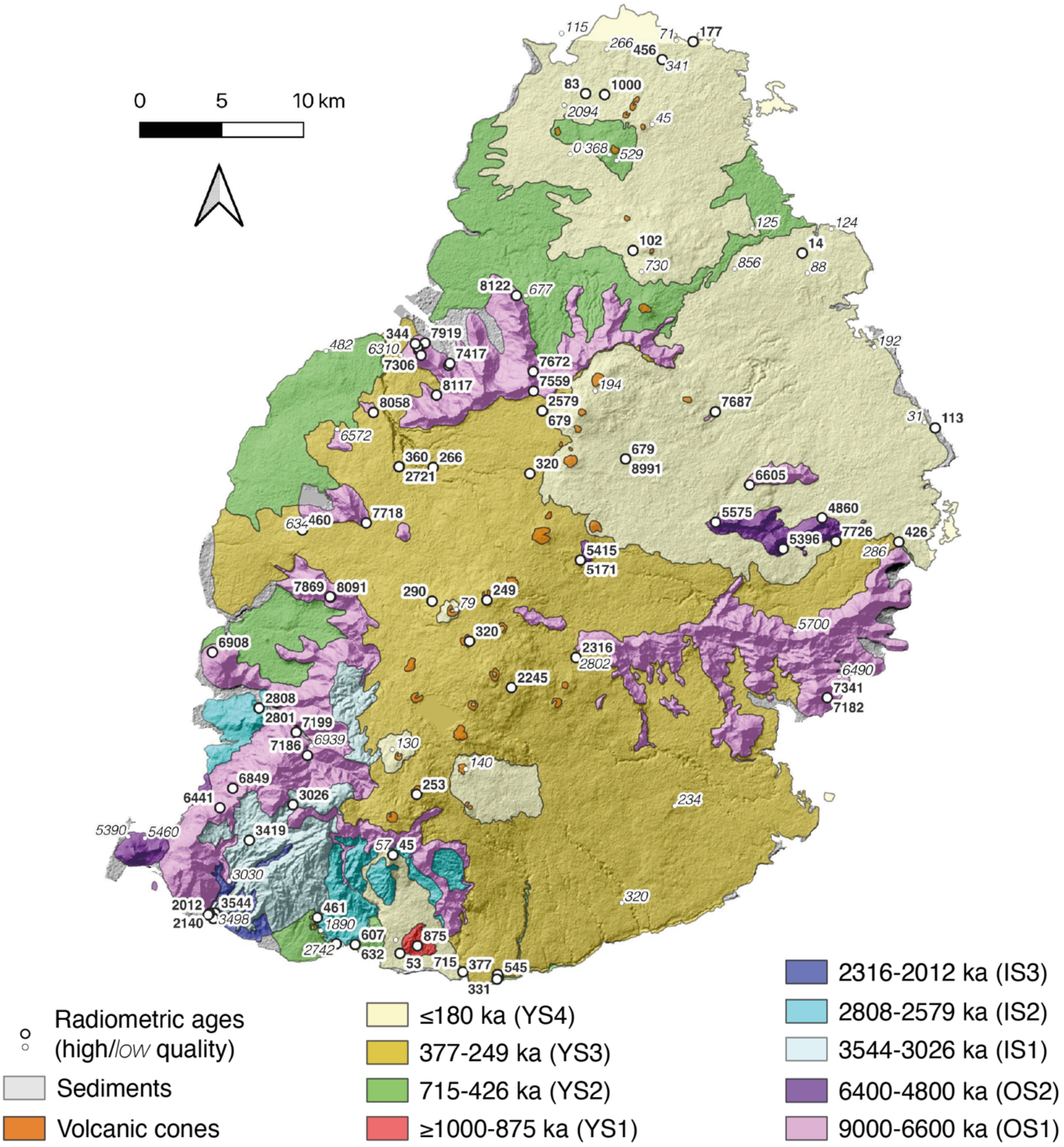

Figure S3: Geological map of Mauritius Island based on field observations, radiometric ages, and geomorphological analysis. OS, Older Series; IS, Intermediate Series; YS, Younger Series. Geology layers are draped on a 30-m-resolution digital elevation model[73].

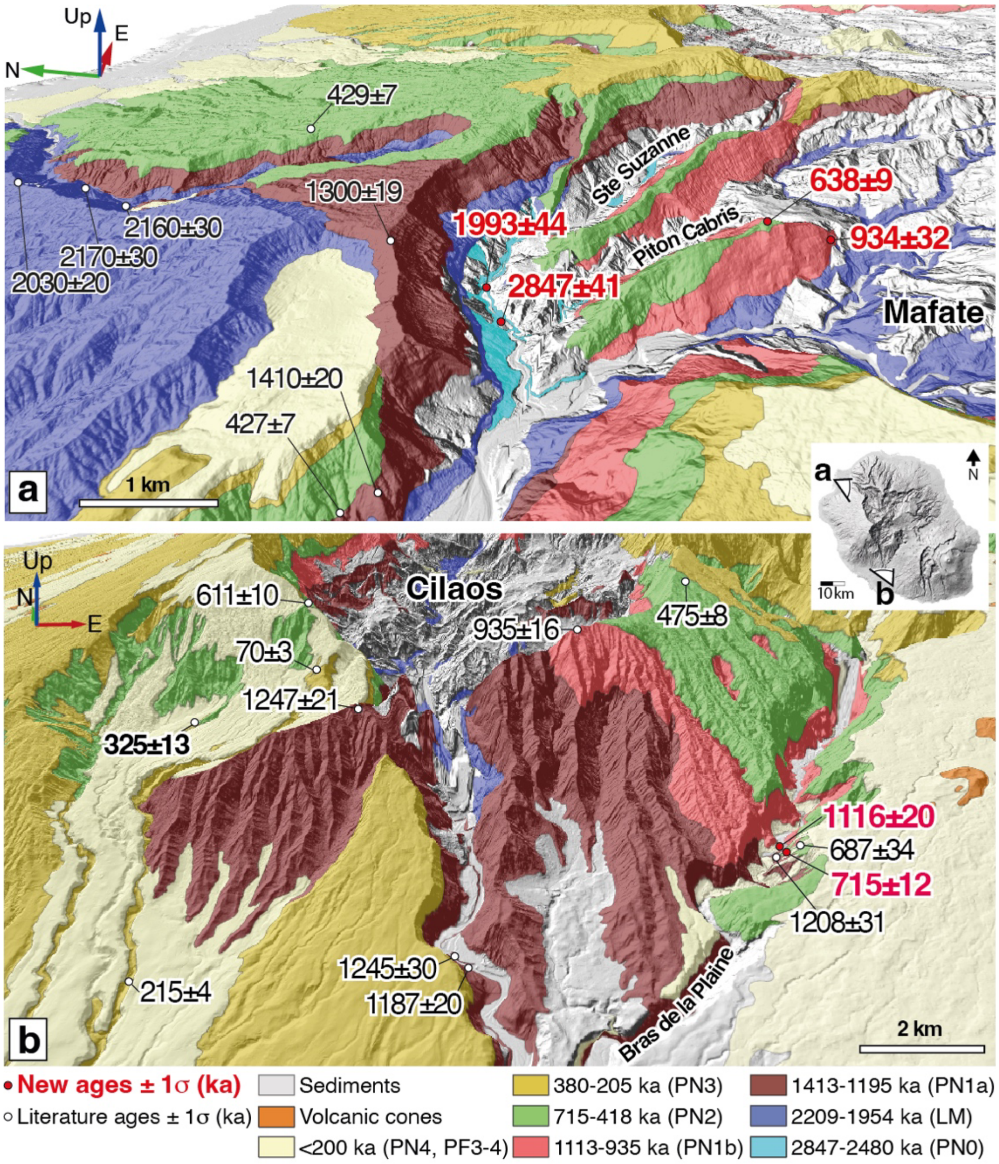

Figure S4: Three-dimensional views of Piton des Neiges, Réunion Island (5 m resolution digital elevation model from the French National Geographic Institute[72], no vertical exaggeration) with our revised subdivision of stratigraphic units based on field mapping, radiometric ages (this study and published data), and geomorphological analysis. a) Northern cirque of Mafate viewed from the west of Réunion Island. b) Bras de La Plaine valley and southern slopes of Piton des Neiges viewed from the south of the island. Note the contrasted erosion patterns among the different chronostratigraphic units, with deeply incised V-shaped valleys in PN1a and progressively shallower valleys in PN1b, PN2, PN3, and PN4 or PF3-4. PN, Piton des Neiges; LM, La Montagne; PF, Piton de la Fournaise. Scale bars apply to the foreground; scale varies with perspective.

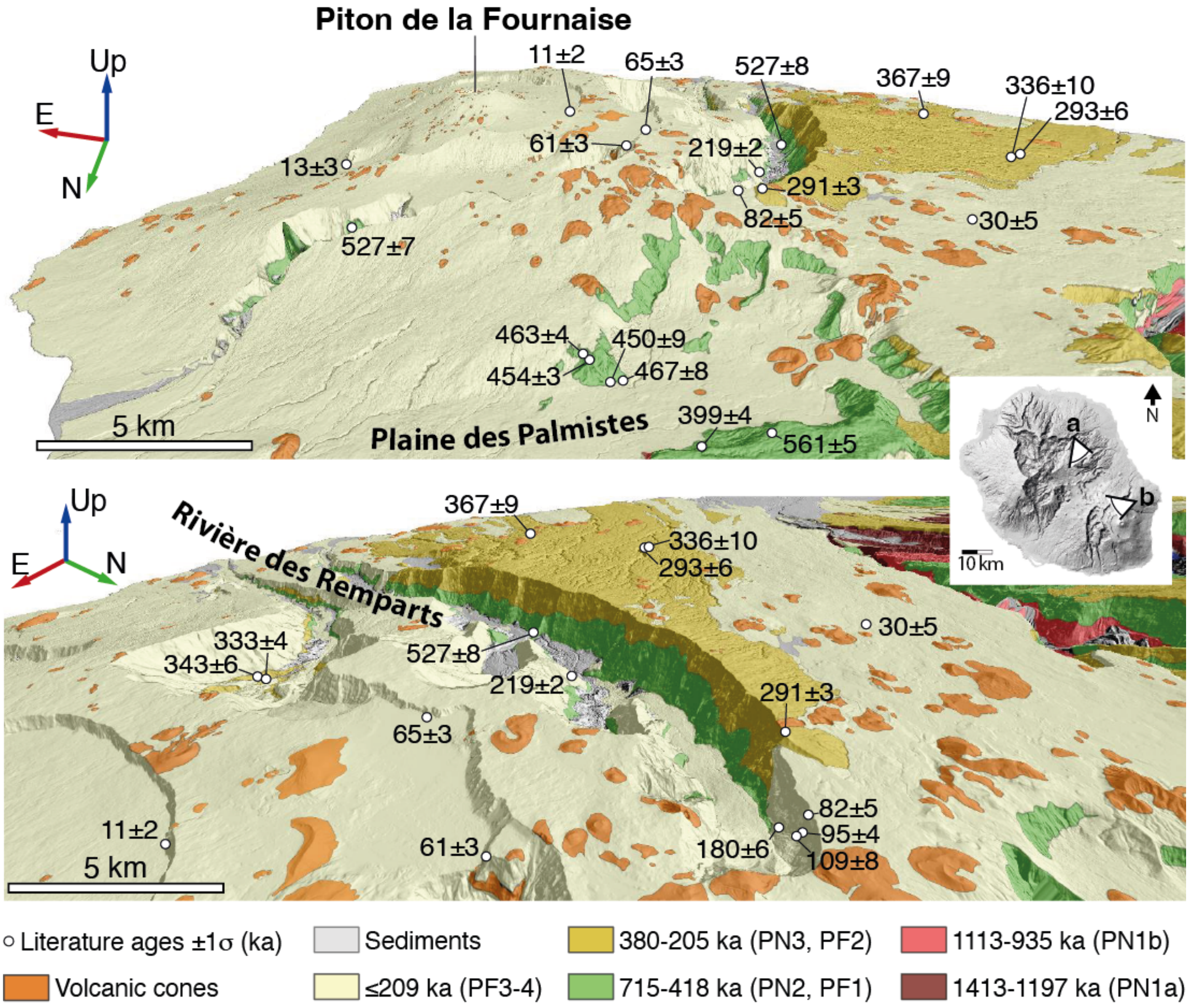

Figure S5: Three-dimensional views of Piton de la Fournaise volcano, Réunion Island (5 m resolution digital elevation model from the French National Geographic Institute[72], x1.5 vertical exaggeration) with our revised subdivision of stratigraphic units, combining field mapping, literature radiometric ages, and geomorphological analysis. a) View from the north of Piton de la Fournaise, showing the erosion contrast between Plaine des Palmistes and the south slopes of the volcano. b) View from the east of the volcano, showing the unconformity in the cliff of the Rivière des Remparts valley. Note the erosion contrast between PF2 and PF3-4. PN, Piton des Neiges; PF, Piton de la Fournaise. Scale bars apply to the foreground; scale varies with perspective.

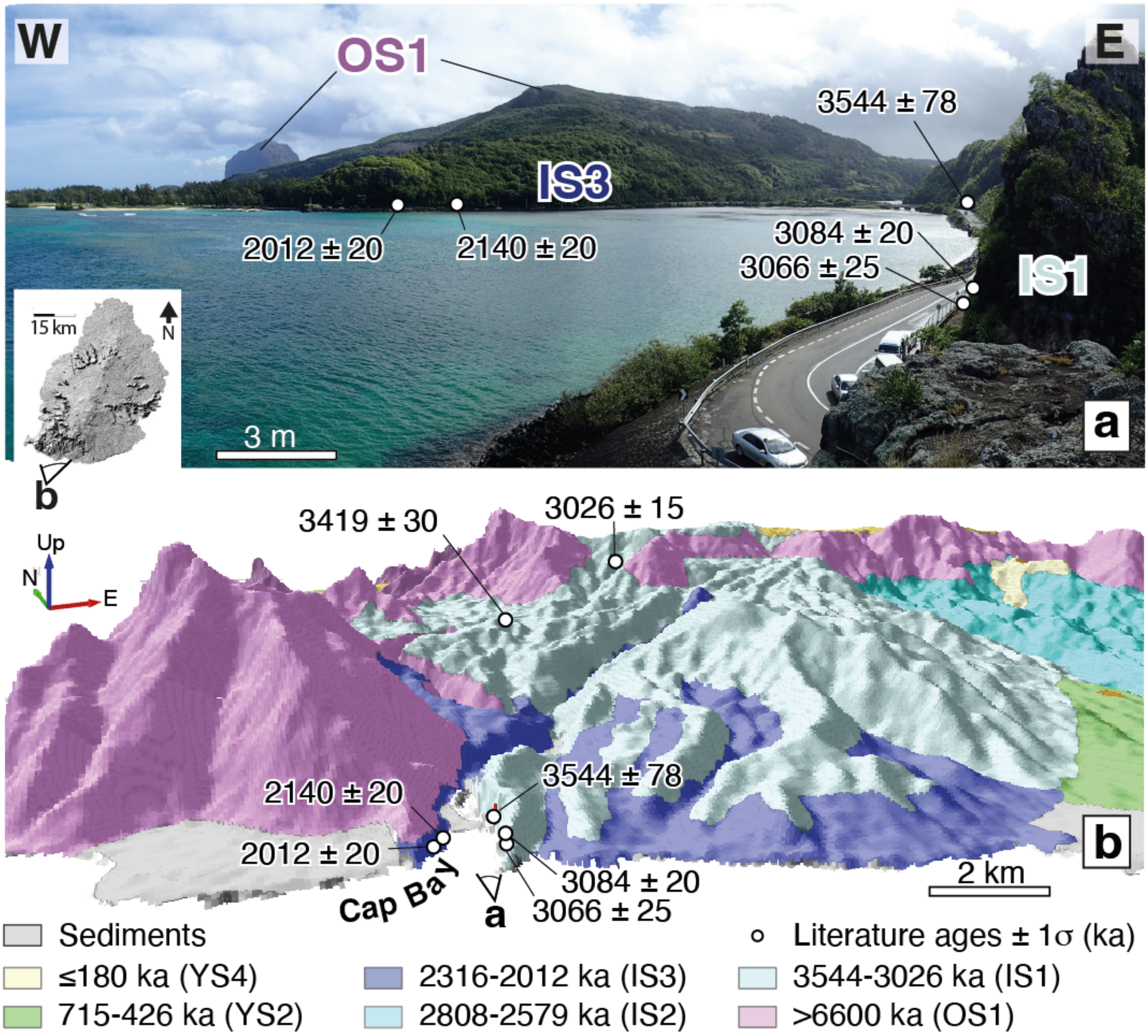

Figure S6: Field (a) and interpreted three-dimensional view (b; SRTM 30 m resolution digital elevation model[73]; x2 vertical exaggeration) of Cap Bay, southern Mauritius, illustrating contrasting morphologies and radiometric ages on the western and eastern sides of the bay. OS, Older Series; IS, Intermediate Series; YS Younger Series. Scale bars apply to the foreground; scale varies with perspective.

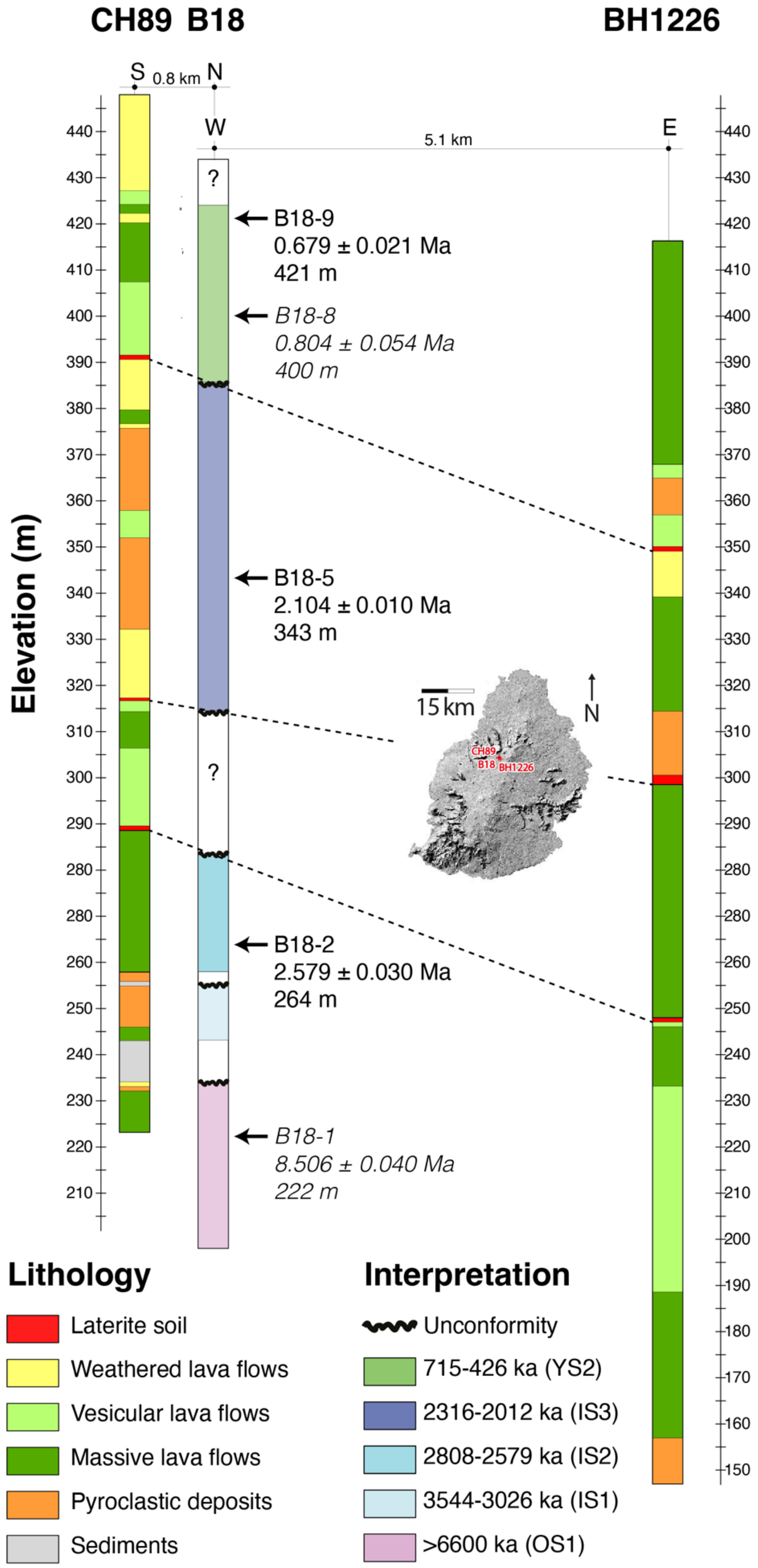

Figure S7: Compilation of borehole data from the Nouvelle Découverte area, central Mauritius (see inset for location, SRTM 30 m resolution digital elevation model[73]), including stratigraphic correlations from boreholes CH89 and BH1226 (ref.[74]) and radiometric ages obtained from drill core samples[32]. Ages shown in italic were rejected in this study. OS, Older Series; IS, Intermediate Series; YS, Younger Series.

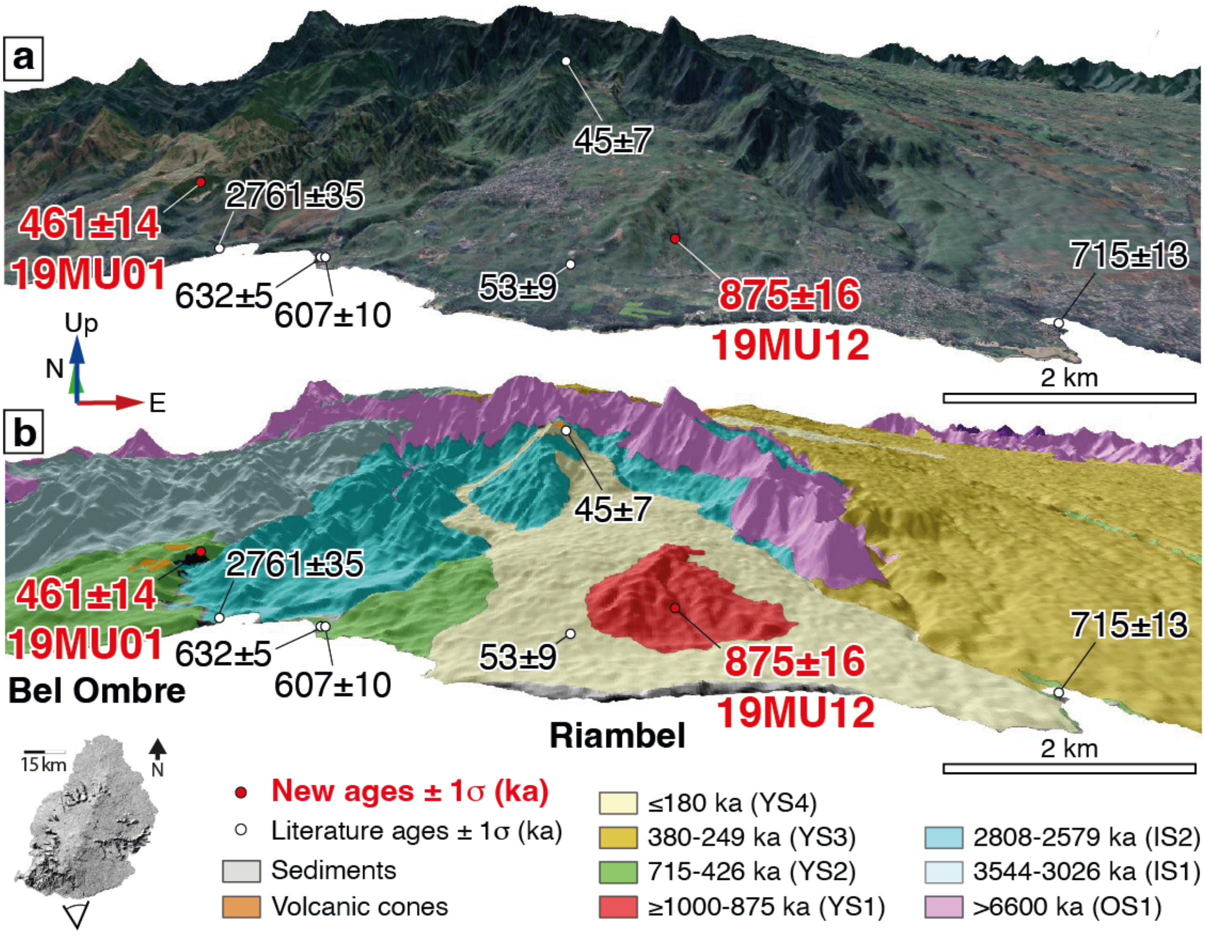

Figure S8: Three-dimensional views of the Bel Ombre and Riambel localities, southern Mauritius (a, Google Image ©2025 Maxar Technologies, data SIO, NOAA, U.S. Navy, NGA, Image ©2025 Airbus; b, geological map, both draped on a 30 m resolution SRTM digital elevation model[73]; x4 vertical exaggeration), showing the top of Riambel's eroded relief dated at 875 ± 16 ka, emerging from a cover of lava flow units aged of 715 ± 13 to 45 ± 7 ka. OS, Older Series; IS, Intermediate Series; YS Younger Series. Scale bars apply to the foreground; scale varies with perspective.

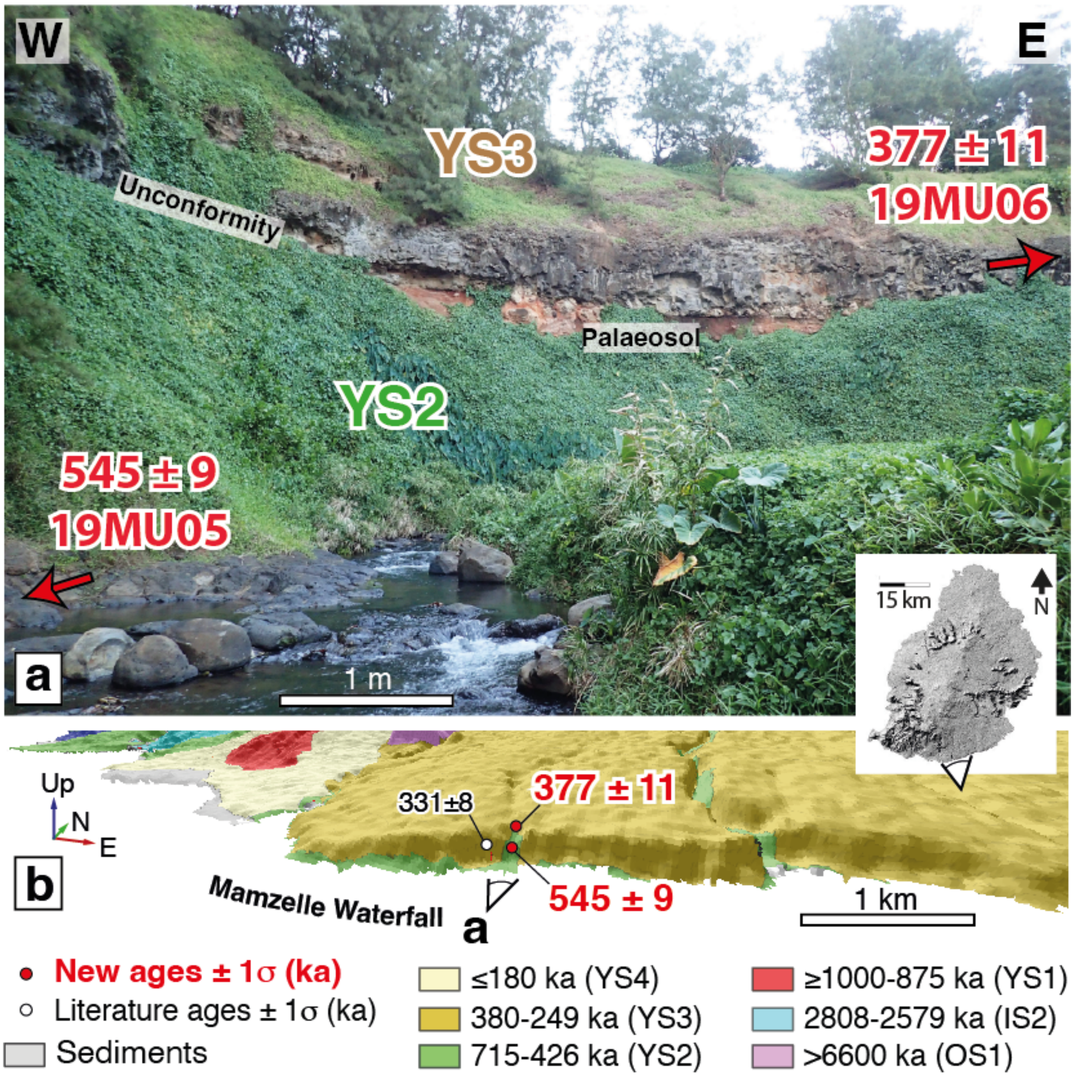

Figure S9: Outcrop view (a) and interpreted three-dimensional view (b; SRTM, 30 m resolution digital elevation model[73]; x2 vertical exaggeration) of the Mamzelle Waterfall valley, southern Mauritius, showing an angular unconformity and palaeosol within the Younger Series bracketed by our two dated samples. OS, Older Series; IS, Intermediate Series; YS, Younger Series. Scale bars apply to the foreground; scale varies with perspective.

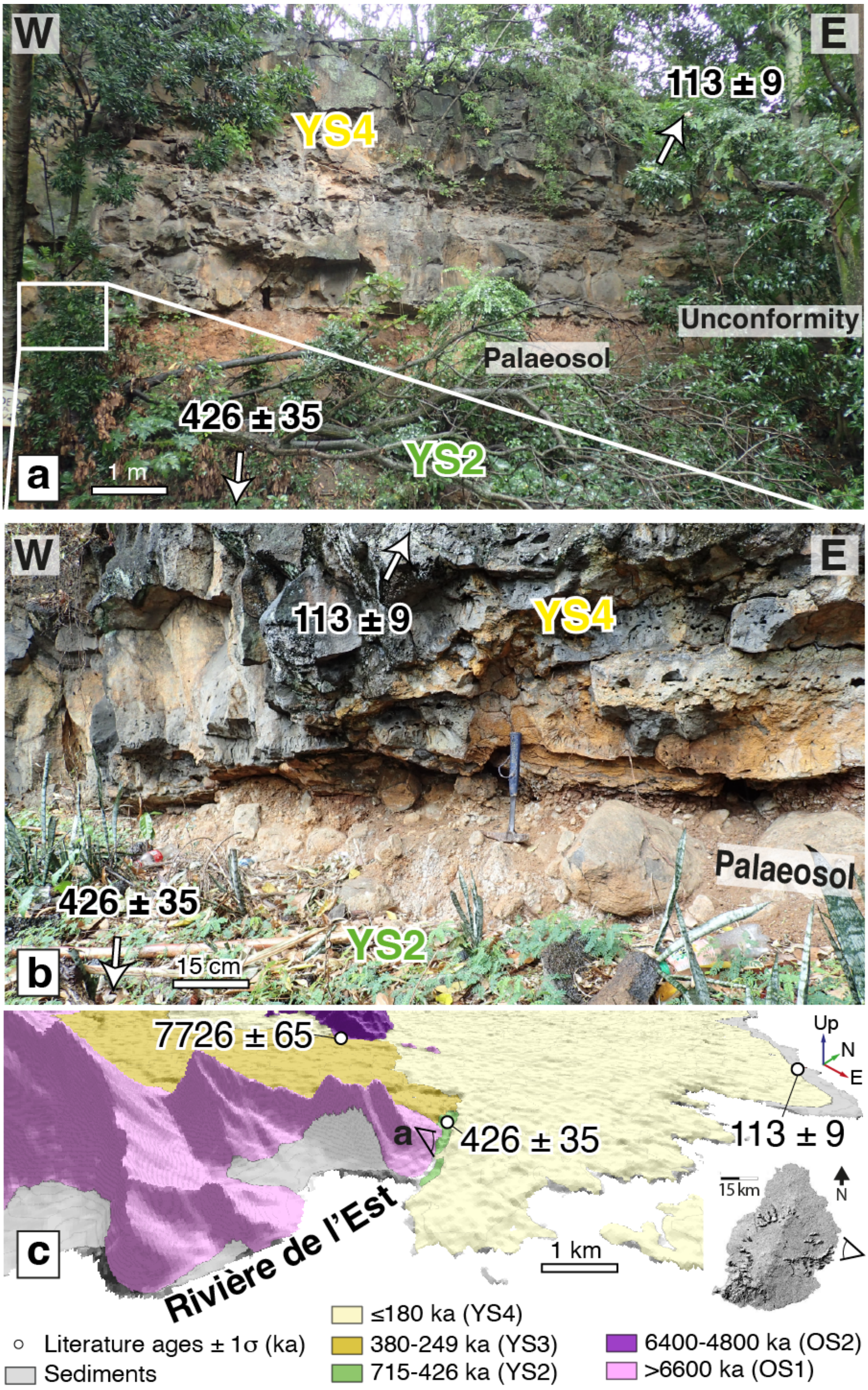

Figure S10: Outcrop views (a, b) and interpreted three-dimensional view (c; SRTM, 30 m resolution digital elevation model[73]; x2 vertical exaggeration) of the Rivière de l'Est valley, eastern Mauritius, showing the angular unconformity between lava flows at 426 ± 35 ka (MU101; ref.[33]) and 113 ± 9 ka (19MU09; ref.[34]). OS, Older Series; IS, Intermediate Series; YS Younger Series. Scale bars apply to the foreground; scale varies with perspective.

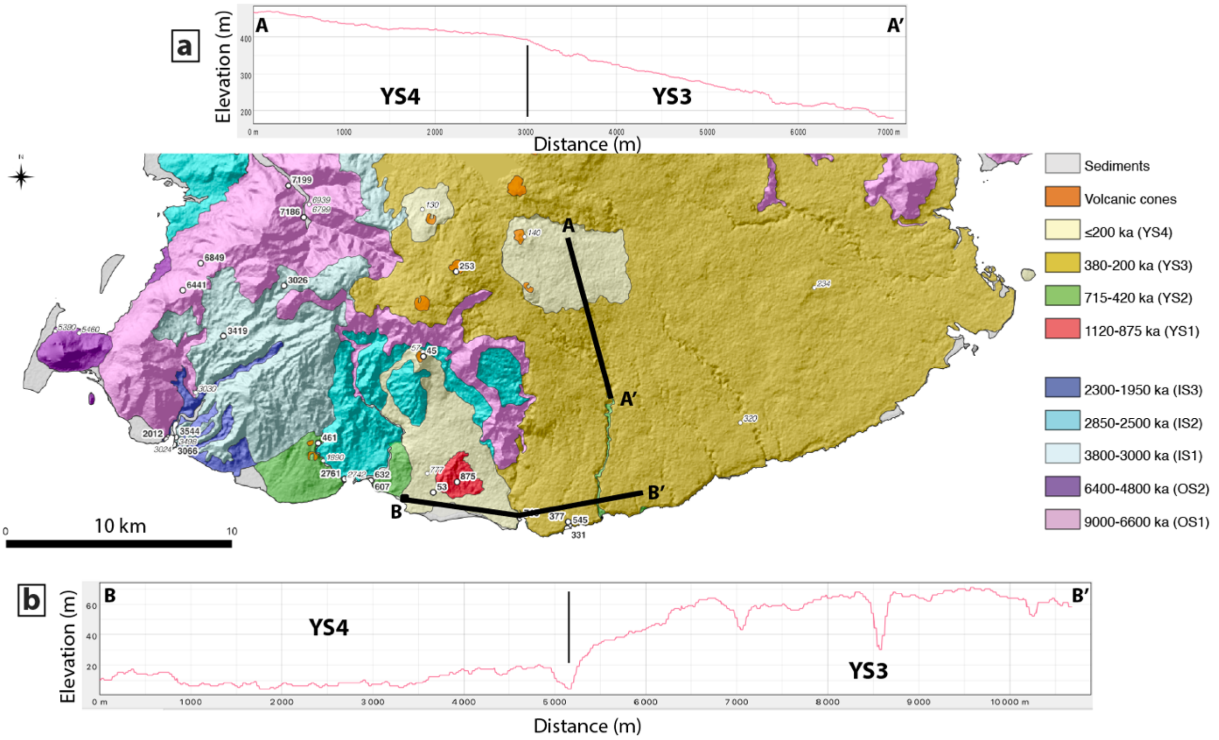

Figure S11: Examples of elevation profiles in southern Mauritius illustrating geomorphic contrasts within the Younger Series (YS). a) Slope change between the smooth YS4 surfaces and the steeper YS3 slopes. b) Erosional contrast between the smooth, weakly incised YS4 surfaces and the more deeply incised YS3 slopes. Map uses the SRTM, 30 m resolution digital elevation model[73].

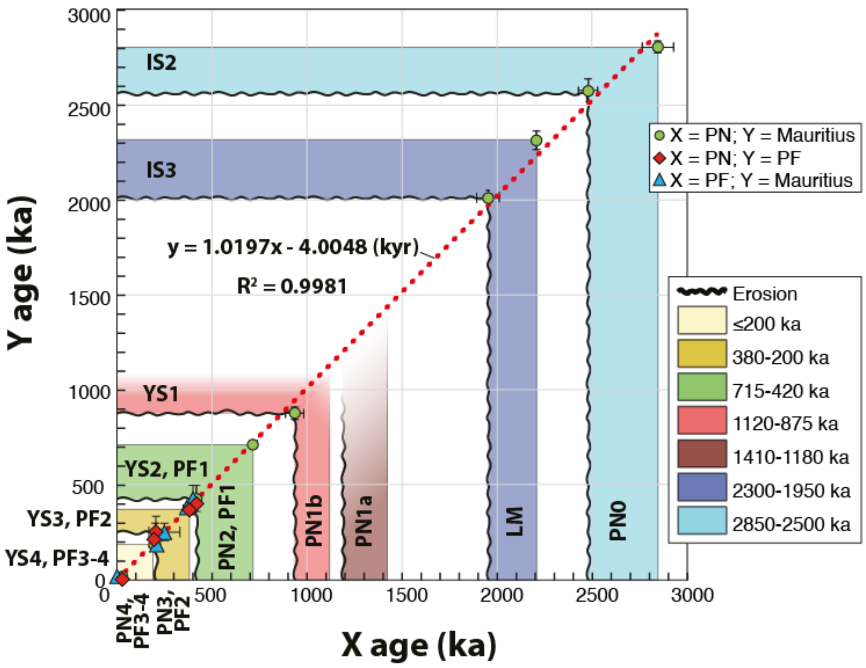

Figure S12: Temporal comparison of volcanic events (onset of constructional phases and erosion intervals) among the three eruptive centres (PN, PF, and Mauritius). Uncertainties are displayed at the 2 SD level for visualization. Linear regression yields a slope and correlation coefficient (R^2) both close to unity, highlighting the synchronicity of volcanism in Réunion and Mauritius.

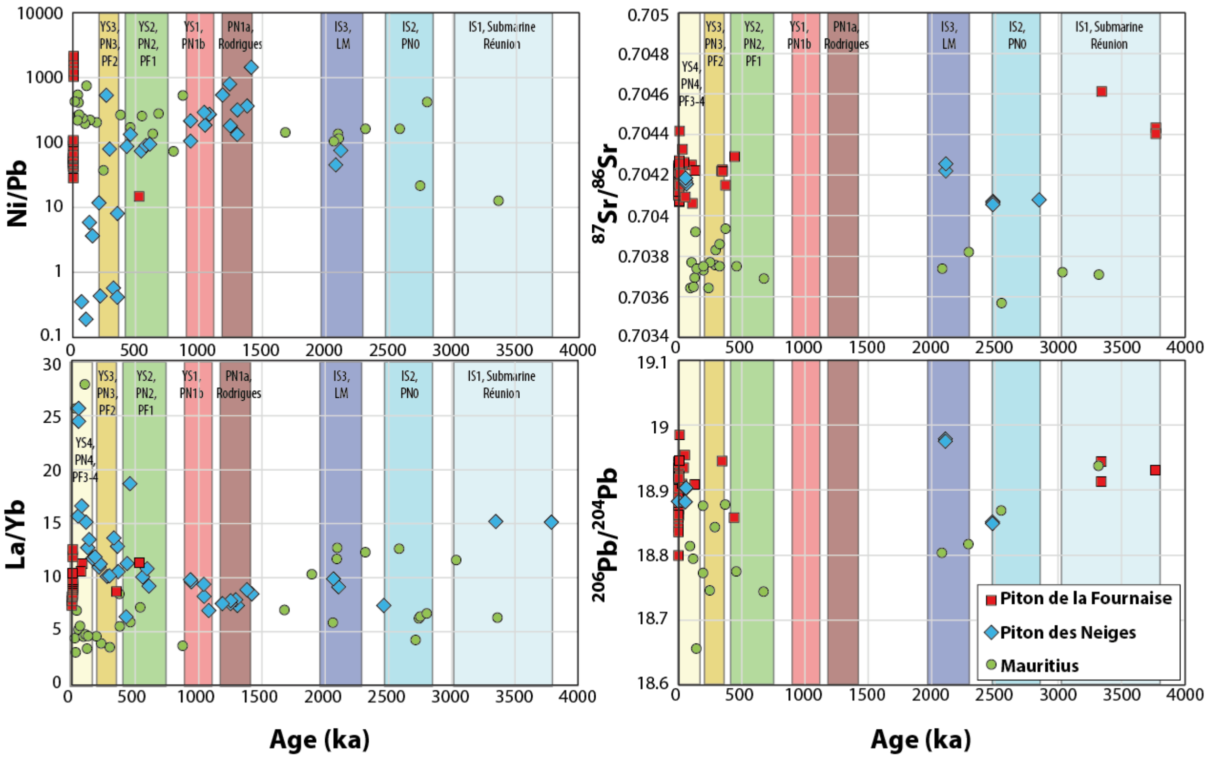

Figure S13: Compilation of trace element ratios and Sr-Pb isotopic data as a function of age for Piton des Neiges (PN), Piton de la Fournaise (PF), and Mauritius (Intermediate Serie, IS; Younger Series, YS), with periods of magmatic activity (Supplementary Data 4, 5). Note the lower Ni/Pb ratios, higher La/Yb ratios, and more radiogenic isotopic compositions of PN and PF magmas compared to those of IS and YS. These features suggest a greater contribution from plume-derived mantle beneath Réunion than beneath Mauritius, potentially associated with lower degrees of partial melting and/or deeper melting conditions.

References cited in the Supplementary Information

1. Kluska, J.-M. Evolution magmatique et morpho-structurale du Piton des Neiges au cours des derniers 500 000 ans. (Université Paris Sud, 1997).
2. Raczek, I., Stoll, B., Hofmann, A. W. & Peter Jochum, K. High-Precision Trace Element Data for the USGS Reference Materials BCR-1, BCR-2, BHVO-1, BHVO-2, AGV-1, AGV-2, DTS-1, DTS-2, GSP-1 and GSP-2 by ID-TIMS and MIC-SSMS. *Geostand. Newsl.* **25**, 77–86 (2001).
3. Gillot, P.-Y., Cornette, Y., Max, N. & Floris, B. Two reference materials, trachytes MDO-G and ISH-G, for argon dating (K-Ar and 40Ar/39Ar) of Pleistocene and Holocene rocks. *Geostand. Geoanalytical Res.* **16**, 55–60 (1992).
4. Germa, A., Quidelleur, X., Labanieh, S., Lahitte, P. & Chauvel, C. The eruptive history of Morne Jacob volcano (Martinique Island, French West Indies): Geochronology, geomorphology and geochemistry of the earliest volcanism in the recent Lesser Antilles arc. *J. Volcanol. Geotherm. Res.* **198**, 297–310 (2010).
5. Fuhrmann, U., Lippolt, H. J. & Hess, J. C. Examination of some proposed K-Ar standards: analyses and conventional K-Ar data. *Chem. Geol. Isot. Geosci. Sect.* **66**, 41–51 (1987).

6. Hess, J. C. & Lippolt, H. J. Compilation of K/Ar measurements on HD-B1 standard biotite; 1994 status report. in *Phanerozoic time scale* vol. 12 19–23 (Bulletin de Liaison et d'information, IUGS Subcommission, Geochronology, 1994).
7. Schwarz, W. H. & Trieloff, M. Intercalibration of 40Ar–39Ar age standards NL-25, HB3gr hornblende, GA1550, SB-3, HD-B1 biotite and BMus/2 muscovite. *Chem. Geol.* **242**, 218–231 (2007).
8. Steiger, R. H. & Jäger, E. Subcommission on geochronology: Convention on the use of decay constants in geo- and cosmochronology. *Earth Planet. Sci. Lett.* **36**, 359–362 (1977).
9. Smietana, M. Pétrologie, géochronologie (K-Ar) et géochimie élémentaire et isotopique (Sr, Nd, Hf, Pb) de laves anciennes de La Réunion: Implications sur la construction de l'édifice volcanique. (Université de La Réunion, 2011).
10. Singer, B. S. *et al.* Precise ages of the Réunion event and Huckleberry Ridge excursion: Episodic clustering of geomagnetic instabilities and the dynamics of flow within the outer core. *Earth Planet. Sci. Lett.* **405**, 25–38 (2014).
11. Baksi, A. K., Hoffman, K. A. & McWilliams, M. Testing the accuracy of the geomagnetic polarity time-scale (GPTS) at 2–5 Ma, utilizing40Ar/39Ar incremental heating data on whole-rock basalts. *Earth Planet. Sci. Lett.* **118**, 135–144 (1993).
12. Baksi, A. K. & Hoffman, K. A. On the age and morphology of the reunion event. *Geophys. Res. Lett.* **27**, 2997–3000 (2000).
13. Quidelleur, X., Holt, J. W., Salvany, T. & Bouquerel, H. New K-Ar ages from La Montagne massif, Réunion Island (Indian Ocean), supporting two geomagnetic events in the time period 2.2-2.0 Ma: New K-Ar ages of the Réunion events. *Geophys. J. Int.* **182**, 699–710 (2010).
14. McDougall, I. The geochronology and evolution of the young volcanic island of Réunion, Indian Ocean. *Geochim. Cosmochim. Acta* **35**, 261–288 (1971).
15. McDougall, I. & Watkins, N. D. Age and duration of the réunion geomagnetic polarity event. *Earth Planet. Sci. Lett.* **19**, 443–452 (1973).
16. Salvany, T. Evolution morphostructurale de volcans boucliers intraplaques océaniques: Exemple des volcans de l'île de la Réunion (Océan Indien). (Université Paris Sud, 2009).
17. Salvany, T., Lahitte, P., Nativel, P. & Gillot, P.-Y. Geomorphic evolution of the Piton des Neiges volcano (Réunion Island, Indian Ocean): Competition between volcanic construction and erosion since 1.4Ma. *Geomorphology* **136**, 132–147 (2012).
18. Cruchet, M., Nehlig, P., Arnaud, N., Chevalier, P. & Lacquement, F. *Nouvelles Datations K-Ar et 14C Dans Le Massif Du Piton Des Neiges*. 87 (2008).
19. Famin, V. La Dynamique du Point Chaud Réunion. (Université de La Réunion, La Réunion, 2018).
20. Gillot, P.-Y. & Nativel, P. K-Ar chronology of the ultimate activity of Piton des Neiges volcano, Reunion Island, Indian ocean. *J. Volcanol. Geotherm. Res.* **13**, 131–146 (1982).
21. Montaggioni, L. F. & Martin-Garin, B. Episodic coral growth events during the building of Reunion and Mauritius shield volcanoes (Western Indian Ocean). *Facies* **66**, 13 (2020).
22. Castellanos Melendez, M. P. *et al.* Explosive volcanism of Piton des Neiges (Reunion Island) and excess age dispersion in sanidine: Insights into magma chamber processes in a hotspot setting. *Chem. Geol.* **632**, 121539 (2023).

23. Famin, V. *et al.* Multitechnique Geochronology of Intrusive and Explosive Activity on Piton des Neiges Volcano, Réunion Island. *Geochem. Geophys. Geosystems* **23**, e2021GC010214 (2022).
24. Raïs, A., Laj, C., Surmont, J., Gillot, P.-Y. & Guillou, H. Geomagnetic field intensity between 70 000 and 130 000 years B.P. from a volcanic sequence on La Réunion, Indian Ocean. *Earth Planet. Sci. Lett.* **140**, 173–189 (1996).
25. Deniel, C., Kieffer, G. & Lecointre, J. New 230Th-238U and 14C age determinations from Piton des Neiges volcano, Reunion — A revised chronology for the Differentiated Series. *J. Volcanol. Geotherm. Res.* **51**, 253–267 (1992).
26. Delibrias, G., Guillier, M.-T. & Labeyrie, J. Gif Natural Radiocarbon Measurements X. *Radiocarbon* **28**, 9–68 (1986).
27. Berthod, C. Relations entre systèmes intrusifs et instabilités sur un volcan basaltique (Piton des Neiges, La Réunion). (Université de La Réunion, La Réunion, 2016).
28. Gillot, P.-Y. & Nativel, P. Eruptive history of the Piton de la Fournaise volcano, Reunion Island, Indian Ocean. *J. Volcanol. Geotherm. Res.* **36**, 53–65 (1989).
29. Gillot, P.-Y., Lefèvre, J.-C. & Nativel, P.-E. Model for the structural evolution of the volcanoes of Réunion Island. *Earth Planet. Sci. Lett.* **122**, 291–302 (1994).
30. Claude, C. *et al.* Timescale of spheroidal weathering of a 293 kyr-old basaltic lava from Réunion Island, Indian Ocean. *Chem. Geol.* **446**, 110–125 (2016).
31. Luais, B. Temporal changes in Nd isotopic composition of Piton de la Fournaise magmatism (Réunion Island, Indian Ocean). *Geochem. Geophys. Geosystems* **5**, 2002GC000502 (2004).
32. Moore, J. *et al.* Evolution of shield-building and rejuvenescent volcanism of Mauritius. *J. Volcanol. Geotherm. Res.* **207**, 47–66 (2011).
33. McDougall, I. & Chamalaun, F. H. Isotopic Dating and Geomagnetic Polarity Studies on Volcanic Rocks from Mauritius, Indian Ocean. *Geol. Soc. Am. Bull.* **80**, 1419 (1969).
34. Quidelleur, X. & Famin, V. Last 150 kyr volcanic activity on Mauritius island (Indian ocean) revealed by new Cassignol-Gillot unspiked K–Ar ages. *Quat. Geochronol.* **82**, 101534 (2024).
35. Ashwal, L. D., Wiedenbeck, M. & Torsvik, T. H. Archaean zircons in Miocene oceanic hotspot rocks establish ancient continental crust beneath Mauritius. *Nat. Commun.* **8**, 14086 (2017).
36. Nohda, S. Systematic Variation of Sr-, Nd- and Pb-Isotopes with Time in Lavas of Mauritius, Reunion Hotspot. *J. Petrol.* **46**, 505–522 (2005).
37. McDougall, I., Upton, B. G. J. & Wadsworth, W. J. A Geological Reconnaissance of Rodriguez Island, Indian Ocean. *Nature* **206**, 26–27 (1965).
38. Vlastélic, I., Staudacher, T. & Semet, M. Rapid Change of Lava Composition from 1998 to 2002 at Piton de la Fournaise (Réunion) Inferred from Pb Isotopes and Trace Elements: Evidence for Variable Crustal Contamination. *J. Petrol.* **46**, 79–107 (2005).
39. Vlastélic, I., Peltier, A. & Staudacher, T. Short-term (1998–2006) fluctuations of Pb isotopes at Piton de la Fournaise volcano (Reunion Island): Origins and constraints on the size and shape of the magma reservoir. *Chem. Geol.* **244**, 202–220 (2007).
40. Vlastélic, I. *et al.* Pb isotope geochemistry of Piton de la Fournaise historical lavas. *J. Volcanol. Geotherm. Res.* **184**, 63–78 (2009).
41. Nauret, F., Famin, V., Vlastélic, I. & Gannoun, A. A trace of recycled continental crust in the Réunion hotspot. *Chem. Geol.* **524**, 67–76 (2019).

42. Pietruszka, A. J., Hauri, E. H. & Blichert-Toft, J. Crustal Contamination of Mantle-derived Magmas within Piton de la Fournaise Volcano, Reunion Island. *J. Petrol.* **50**, 661–684 (2009).
43. Paul, D., White, W. M. & Blichert-Toft, J. Geochemistry of Mauritius and the origin of rejuvenescent volcanism on oceanic island volcanoes. *Geochem. Geophys. Geosystems* **6**, 2004GC000883 (2005).
44. Ashwal, L. *et al.* A Mantle-derived Origin for Mauritian Trachytes. *J. Petrol.* egw052 (2016) doi:10.1093/petrology/egw052.
45. Newsom, H. E., White, W. M., Jochum, K. P. & Hofmann, A. W. Siderophile and chalcophile element abundances in oceanic basalts, Pb isotope evolution and growth of the Earth's core. *Earth Planet. Sci. Lett.* **80**, 299–313 (1986).
46. Fisk, M. R., Upton, B. G. J., Ford, C. E. & White, W. M. Geochemical and experimental study of the genesis of magmas of Reunion Island, Indian Ocean. *J. Geophys. Res. Solid Earth* **93**, 4933–4950 (1988).
47. Albarede, F. & Tamagnan, V. Modelling the Recent Geochemical Evolution of the Piton de la Fournaise Volcano, Reunion Island, 1931-1986. *J. Petrol.* **29**, 997–1030 (1988).
48. Graham, D., Lupton, J., Albarède, F. & Condomines, M. Extreme temporal homogeneity of helium isotopes at Piton de la Fournaise, Réunion Island. *Nature* **347**, 545–548 (1990).
49. Fretzdorff, S. & Haase, K. M. Geochemistry and petrology of lavas from the submarine flanks of Réunion Island (western Indian Ocean): implications for magma genesis and the mantle source. *Mineral. Petrol.* **75**, 153–184 (2002).
50. Bosch, D. *et al.* Pb, Hf and Nd isotope compositions of the two Réunion volcanoes (Indian Ocean): A tale of two small-scale mantle "blobs"? *Earth Planet. Sci. Lett.* **265**, 748–765 (2008).
51. Albarede, F. *et al.* The Geochemical Regimes of Piton de la Fournaise Volcano (Reunion) During the Last 530 000 Years. *J. Petrol.* **38**, 171–201 (1997).
52. Vigouroux, N., Williams-Jones, A. E., Wallace, P. & Staudacher, T. The November 2002 eruption of Piton de la Fournaise, Réunion: tracking the pre-eruptive thermal evolution of magma using melt inclusions. *Bull. Volcanol.* **71**, 1077–1089 (2009).
53. Vlastélic, I. *et al.* Origin and fate of sulfide liquids in hotspot volcanism (La Réunion): Pb isotope constraints from residual Fe–Cu oxides. *Geochim. Cosmochim. Acta* **194**, 179–192 (2016).
54. Di Muro, A. *et al.* The Shallow Plumbing System of Piton de la Fournaise Volcano (La Reunion Island, Indian Ocean) Revealed by the Major 2007 Caldera-Forming Eruption. *J. Petrol.* **55**, 1287–1315 (2014).
55. Valer, M. Origine et évolution des magmas de l'île de la Réunion: apports de la pétro-géochimie et des inclusions magmatiques. (Clermont-Ferrand, 2016).
56. McDougall, I. & Compston, W. Strontium Isotope Composition and Potassium–Rubidium Ratios in some Rocks from Réunion and Rodriguez, Indian Ocean. *Nature* **207**, 252–253 (1965).
57. Oversby, V. M. Genetic relations among the volcanic rocks of Réunion: chemical and lead isotopic evidence. *Geochim. Cosmochim. Acta* **36**, 1167–1179 (1972).
58. Ludden, J. N. Magmatic evolution of the basaltic shield volcanoes of Reunion Island. *J. Volcanol. Geotherm. Res.* **4**, 171–198 (1978).

59. Dupré, B. & Allègre, C. J. Pb–Sr isotope variation in Indian Ocean basalts and mixing phenomena. *Nature* **303**, 142–146 (1983).
60. Vlastélic, I. *et al.* Lead isotopes behavior in the fumarolic environment of the Piton de la Fournaise volcano (Réunion Island). *Geochim. Cosmochim. Acta* **100**, 297–314 (2013).
61. Duncan, R. A., Quilty, P. G., Barling, J. & Fox, J. M. Geological development of Heard Island, Central Kerguelen Plateau. *Aust. J. Earth Sci.* **63**, 81–89 (2016).
62. Verwoerd, W. J. *et al.* F. Oceanic Islands on the Antarctic Plate. in *Antarctic Research Series* (eds LeMasurier, W. E. et al.) vol. 48 396–463 (American Geophysical Union, Washington, D. C., 1990).
63. McDougall, I., Verwoerd, W. & Chevallier, L. K–Ar geochronology of Marion Island, Southern Ocean. *Geol. Mag.* **138**, 1–17 (2001).
64. Fox, J. M., McPhie, J., Carey, R. J., Jourdan, F. & Miggins, D. P. Construction of an intraplate island volcano: The volcanic history of Heard Island. *Bull. Volcanol.* **83**, 37 (2021).
65. Segard, M. Pétrogénèse des laves de l'ïle de la Possession (archipel de Crozet) et implications pour les hétérogénéités lithologiques des sources de points chauds. (2013).
66. Lameyre, J. *et al.* Chronological evolution of he Kerguelen islands syenite-granite ring complex. *Nature* vol. 263 306–307 (1976).
67. Weis, D., Frey, F. A., Giret, A. & Cantagrel, J.-M. Geochemical Characteristics of the Youngest Volcano (Mount Ross) in the Kerguelen Archipelago: Inferences for Magma Flux, Lithosphere Assimilation and Composition of the Kerguelen Plume. *J. Petrol.* **39**, 973–994 (1998).
68. Nougier, J. Geochronology of the volcanic activity on Îles Kerguelen. in *Antarctic Geology and Geophysics* (ed. Adie, R. J.) 803–808 (Universitetsforlaget, Oslo, 1972).
69. Chevallier, L., Nougier, J. & Cantagrel, J. M. Volcanology of Possession Island, Crozet Archipelago (TAAF). in *Antarctic Earth Science* (eds Oliver, R. L., James, P. R. & Jago, J. B.) 652–658 (Cambridge University Press, New York, 1983).
70. Gagnevin, D. *et al.* Open-system processes in the genesis of silica-oversaturated alkaline rocks of the Rallier-du-Baty Peninsula, Kerguelen Archipelago (Indian Ocean). *J. Volcanol. Geotherm. Res.* **123**, 267–300 (2003).
71. Quilty, P. G., Murray-Wallace, C. V. & Whitehead, J. M. *Austrochlamys heardensis* (Fleming, 1957) (Bivalvia: Pectinidae) from Central Kerguelen Plateau, Indian Ocean: palaeontology and possible tectonic significance. *Antarct. Sci.* **16**, 329–338 (2004).
72. Institut Géographique National. RGE ALTI® 5M - D974 La Réunion - Septembre 2023. (2023).
73. Earth Resources Observation and Science (EROS) Center. Shuttle Radar Topography Mission (SRTM) 1 Arc-Second Global. U.S. Geological Survey https://doi.org/10.5066/F7PR7TFT (2000).
74. Romagnoli, P., Pisani, P., Pasqua, C. & Mazzoleni, G. *Opportunity Assessment for the Development of Geothermal Energy in Mauritius*. 34PP plus 2 annexes (2015).
