# Supplementary material for: Synchronized Mascarene volcanism reveals 400 kyr cycles in melt supply from the Réunion plume
Source: Nat Commun. 2026 May 7;17:6118. doi: 10.1038/s41467-026-72855-1 (PMC13358134; doi:10.1038/s41467-026-72855-1)
Supplement: Supplementary file 2 — Description of Additional Supplementary Files [file 41467_2026_72855_MOESM2_ESM.pdf]

Description of Additional Supplementary Files

Supplementary Data 1: K–Ar ages from this study. Column headings include sample names; latitude and longitude (decimal degrees); potassium (K) concentration (wt%); concentration of radiogenic argon ($^{40}Ar^*$; %); concentration of $^{40}Ar^* \times 10^{11}$ (atoms per gram); age (ka); 1σ uncertainty (ka); weighted mean age (ka); and 1σ weighted mean uncertainty (ka).

Supplementary Data 2: Compilation and filtering of literature radiometric ages for the Mascarene Islands. Data for Piton des Neiges volcano are from refs[1,9–27]. Data for Piton de la Fournaise are from refs[1,9,14,19,28–31]. Ages for Mauritius and Rodrigues are from refs[21,32–37].

Supplementary Data 3: Summary of geological observations and radiometric ages used to subdivide the history of the Mascarene Islands into chronostratigraphic units.

Supplementary Data 4: Compilation of trace element data combined with radiometric ages (Supplementary Data 2) from the Mascarene Islands. Data are from refs[9,14,16,18,32,34,36,38–44].

Supplementary Data 5: Compilation of Sr-Nd-Hf-Pb isotopic data combined with radiometric ages (Supplementary Data 2) from the Mascarene Islands. The isotopic database and filtering procedure follow Nauret at al.[41]. Data are from refs[9,31,32,36,38–60].

Supplementary Data 6: Compilation of chronostratigraphic charts for Marion and Prince Edward Islands, Possession and East Islands (Crozet Archipelago), the Kerguelen Archipelago, and Heard Island over the Upper Miocene and Quaternary. Sources are from refs[61–71].
