# Supplementary material for: Synchronized Mascarene volcanism reveals 400 kyr cycles in melt supply from the Réunion plume
Source: Nat Commun. 2026 May 7;17:6118. doi: 10.1038/s41467-026-72855-1 (PMC13358134; doi:10.1038/s41467-026-72855-1)
Supplement: Supplementary file 9 — Transparent Peer Review file [file 41467_2026_72855_MOESM9_ESM.pdf]

nature portfolio

Peer Review File

Synchronized Mascarene volcanism reveals 400 kyr cycles in melt supply from the Réunion plume

Corresponding Author: Professor Vincent Famin

This file contains all reviewer reports in order by version, followed by all author rebuttals in order by version.

Version 0:

Reviewer comments:

Reviewer #1

(Remarks to the Author)
Revision report of NatComms NCOMMS-25-43481 manuscript:

Short-period (~400 kyr) melting pulsation of the Réunion plume, by Famin et al 2025.

I'm including a revised pdf file and a general review report for this manuscript, please se below:

Key Results

The manuscript uses field mapping, geomorphology and K-Ar geochronology to define a magma production time for the Reunion hotspot, from La Reunion and Mauritius islands. Specifically, they analyze more than 400 published K-Ar ages and performed new radiometric ages (10, also K-Ar). With these data they conclude that the hotspot responsible for producing melt batches at this specific location worked in pulses of around 400 000 years (400 kyr).

Validity

Although the data was filtered, analyzed, organized and plotted against their own, the study has some limited original data, but makes a good use of the available voluminous data set in terms of geochronology.

The document provides field evidence, using DEM's, maps, drill-core data and geomorphological interpretations. As well as some field photos in the supplemental file. I found, however, that the general use of the geomorphological features is limited and not used in a thorough way. The authors only describe landscape aspects in a general form, not very concise and lacking some thorough revision of these features.

Significance

The findings are important and significant for an audience interested in hotspot volcanism, ocean island volcanism, magma production, evolution and periodicity. The main finding (relatively short 400 kyr eruption periods/clusters) do not contradict previous studies or puts it in conflict. However, the data available do not provide a very direct link to these observations.

Data and methods

The manuscript contains three main sets of data: 1) field data; 2) geomorphological assessment; 3) K-Ar geochronology, published and performed by the authors.

Field data is well presented in terms of geological (lithological) maps of the islands, generalized stratigraphic columns, and cross sections in DEM's and drill core data logs. The geomorphological interpretations come from DEM's, one with 5 m resolution but the other with 30 m resolution, which is relatively low resolution to provide good geomorphological interpretations in such area. And finally, the K-Ar data: these is a robust data set, but mostly from the literature, it has been filtered and processed for quality control and correspond to around 400 data points organized in kyrs at a one sigma confidence level. However, if we compare this, with the original data, only 10 data points, it's relatively small.

The figures and tables are well presented in general, with some specific comments directly to them to improve the readability.

Analytical approach

The analytical method used for the K.Ar age determinations looks thorough and comprehensive, but this applies mostly for their own samples and performed dates. The problem is the control over these other 300 samples used to complete the model proposed, where they didn't have the control on those samples.

The geological approach is relevant, but the geomorphological approach is the one that is not very well developed throughout the document. Some of the geomorphological interpretations seem slightly vague and general. For example, in the use of concepts like "inverted topography", unconformities, breccias, sediments, scarps, paleosols, and other features that could be much better described/interpreted to align to the findings, or not.

Suggested improvements

General overview
• The manuscript is appropriately structured, contains original and published data, the figures are of good quality and the data tables and supplemental information is provided. The authors explain their methods in terms of the K-Ar dating, the reason of using this approach and mention something about the geological, stratigraphic and geomorphological approach.
• The geomorphological approach is the one that in my opinion is less well developed and it lacks a precise and ordered scheme. They use DEM's but at different resolutions, include some terms but used in a relatively vague manner.
• In terms of the data, the document is complete.
• The text is well written but somehow difficult to follow, mostly because the use of too many acronyms, several ages that interrupt the flow of the text, and complex sample numbers between sentences and phrases.
• The conclusions reached could be supported by the data, but mostly the geochronology, not much support from the geomorphological approach in this aspect.
• At the end, the discussions refer mostly to deep mantle magma dynamics, using several published studies and coupling it with their interpretations. This is fair, but the discussion should also refer to the other aspects worked in the manuscript like the geology/stratigraphy and geomorphology.

Specific points:

- A pdf is attached with specific points that require attention to improve the quality of the manuscript.
- Although not distracting from conclusions, the written manuscript could be improved by the revision of a native English speaker/writer.
- The title is short and could appear slightly misleading or not informative enough. I would suggest including more information without extending it too much.

Finally, the manuscript makes good use of references, cites and refers to updated and classic literature and provides recognition to previous interpretations.

Reviewer #2

(Remarks to the Author)
I support publication pending revision.

This manuscript presents a detailed reconstruction of short-period magmatic fluctuations (approximately 400 kyr) at the Réunion hotspot over the last 3 million years, based on high-resolution chronostratigraphy from Réunion and Mauritius islands. The study combines field mapping, geomorphological observations, and an extensive compilation of radiometric age data, including 10 new K-Ar dates, to propose a rhythmic volcanic tempo that may reflect plume-related melt pulses. The integration of geomorphology, geology, and geochronology provides a well-resolved view of the volcanic history of these islands. The authors present a revision of volcanic chronostratigraphies for both Réunion and Mauritius, significantly improving upon previous models. Particularly interesting is the identification of synchronous volcanic activity between the two islands, which are separated by approximately 230 kilometers. The manuscript also includes a discussion of potential mechanisms behind this synchronicity, addressing plume dynamics and melt migration scenarios in a comprehensive and convincing manner. Furthermore, the authors draw a compelling connection to similar magmatic periodicity in the Deccan Traps, suggesting a possible long-lived pulsation signal of the Réunion plume.
In summary, this paper presents a rich and well-argued case for short-period magmatic pulsation in the Réunion hotspot and its implications for mantle plume dynamics. However, additional clarification of the methodology, broader context through comparison with other hotspots (particularly Hawaii), and careful distinction between generation and migration of melts would substantially strengthen the manuscript.
GENERAL COMMENTS.
1. The concluding suggestion that approximately 400 kyr magmatic pulsation may be a global feature is intriguing but underdeveloped. The case of Hawaii, where similar datasets (such as dozens to hundreds of high-precision U-Th/He and

Ar-Ar ages) exist, is not discussed. Are there similar periodicities observed there, or is Réunion truly unique? Without this comparison, the claim of global relevance remains speculative.

2. In lines 70-71: The authors refer to the Deccan Traps as a suspected cause of the Cretaceous–Paleogene mass extinction, citing voluminous flood basalt eruptions. However, the role of the Chicxulub impact as a primary or at least co-contributing factor in the extinction event is widely accepted. The authors should acknowledge the complexity of this debate and clarify that current consensus generally considers a combination of both impact and volcanism as drivers of the extinction, rather than attributing it solely to Deccan volcanism.

3. In line 133, the manuscript notes that some units, such as Rodrigues, are constrained only by two K-Ar ages published before the development of modern dating techniques. However, the present study also relies primarily on the K-Ar method. This raises a conceptual inconsistency: if earlier K-Ar ages are considered outdated, why was the same technique chosen for new data acquisition? The authors justify their use of K-Ar for young mafic rocks, but it remains unclear why more advanced or complementary techniques, such as Ar-Ar, U-Pb, or U-Th/He, were not employed. Given the emphasis on high-resolution temporal patterns, a discussion of the methodological limitations and advantages of K-Ar compared to modern alternatives is warranted.

4. While the observed synchrony of volcanic activity between Réunion and Mauritius is interesting, the interpretation that this pattern necessarily reflects periodic melt generation deep in the mantle plume may be premature. Alternative explanation, such as episodic magma storage and release, could also produce a similar volcanic signal. The authors are encouraged to clarify whether the ~400 kyr periodicity reflects the timing of melt generation at depth or rather the dynamics of melt transport and eruption. A clearer distinction between these processes: melt generation versus melt delivery, would strengthen the interpretation of the underlying geodynamic mechanism.
5. The authors cite general references to 200–400 kyr activity phases in other intraplate volcanic settings, but this should be expanded into a more substantive comparison, possibly as a table or extended figure in the supplementary material, to strengthen the broader relevance of the findings.

Reviewer #3

(Remarks to the Author)
Review of manuscript ‘Short-period (~400 kyr) melting pulsation of the Réunion plume” submitted by Famin et al. for publication in Nature Communications

I read with great interest the manuscript submitted by Famin et al. for publication in Nature Communications. In their study, the authors address the overall question of pulsation of mantle plumes based on geological, geochronological and geomorphological evidence from la Reunion and Mauritius Islands. Based on a compilation of existing geochronological data and several new dates of different phases of activities on these two islands, on evidence of erosional discontinuities between these phases and on concordance of active and inactive periods across the two islands, the authors argue for evidence of ‘short-term’ (~400 kyr) pulsation of the Reunion mantle plume. The proposed hypothesis is quite novel as it suggests much shorter term fluctuation of hot spot volcanism compared to previously described variations of the order of several million years.
The manuscript is well structured and illustrated by a set of well-designed figures: after introducing the general research question around pulsation of mantle plumes, they present geochronological and geomorphological evidence for a selection of key sections on Reunion and Mauritius Islands. The 3D representations of their geological interpretations coupled with the compilation of existing geochronological data and addition of several new ages for key activity phases are great contributions of this study. Figure 6 is then presenting the core contribution of the paper, highlighting the similar timing in phases of activity and repose for both islands associated to the Reunion hotspot, despite the distance separating them. This study is of broad scientific interest and is based on solid evidence. It is therefore suitable for publication in a highly ranked journal such as Nature communications.
I have two related remarks that could be considered by the authors when revising their publication:
- The authors described a few geological sections in which several phases of volcanic activity are clearly separated by erosion discontinuities. Based on similar dates for the early and late lava flows of each phase at these different locations, they make the fundamental hypothesis that the two islands evolved through island-wide constructional phases separated by island-wide erosional phases. This hypothesis is mentioned explicitly on line 258 ‘considering these erosion events as island-wide episodes’, but is made implicitly throughout. If one consider the current evolution of Piton de la Fournaise, we see active accumulation of lava flows over the last decades/centuries in some sectors, with concomitant development of deep erosional canyons in others. This suggests, as observed on other volcanoes where topography or deformation concentrate eruptive products on some flanks, that constructional and erosional processes can be intimately intertwined on volcanoes and that differentiation might be more spatial than temporal (especially on large shields for which eruptive activity can concentrate in some sectors due to stress field evolution).
While I understand that the locations where stratigraphic evidence can be studied are limited by the availability of sections created by erosion, authors should further discuss their rational for assuming that the sequences (both phases of activity and erosion) documented are representative for the entire island. And if there are, is it possible to reconstruct the approximate morphology of the island at these different stage of evolution, in order to estimate the volume of magma associated with each phase?
- Associated with the previous point, figure 6 highlights that the period of repose between the different phases of activity tend

to decrease with time, with hiatus of less than 50.000 years between the 3 phases of activity defined over the last million years at the three volcanoes considered. One would wonder if these more limited evidence of absence of activity in the recent period cannot be linked to the fact that recent volcanic deposits are outcropping over more areas of the islands, have been less affected by erosion and have been assessed by more dating. By consequence, we could question whether the discrete phases of activity separated by long hiatus for the period over 1Myr are not largely due to the limited outcropping of these phases, the destruction of some deposits by erosion and the limited number of available dating.
I do not mean that the main point made by the authors is not valid, but these limitations, which are intrinsic to the study of the geological stratigraphy of volcanoes based on field evidence, should be acknowledge and discussed by authors. Authors could also consider whether the pulsation of the plume should be leading to full cessation of volcanic activity or rather to fluctuation of the volumetric output rate over time, and whether this volumetric output rate through time can be reconstructed based on geological evidence (especially for the most recent phases with more widespread outcrops).

Minor comments:
- Line 88 : ‘two latest volcanic edifices’. Authors refer to each island as one volcanic edifices, whereas Reunion Island is build from at least two volcanoes (PN and PF). Terminology used should be clarified.
- Lines 178-187 : dates reported in that paragraph do not match the age limits of the classes used in figure 3. Especially for base of PN1b, the date of 1116 is conflicting with the limit of 1113 ka. If there is a reason for these differences, it should be explained.

Version 1:

Reviewer comments:

Reviewer #1

(Remarks to the Author)
Review report of revised version of Manuscript#: NCOMMS-25-43481A by Fanin and collaborators for Nature Communications.

The revised manuscript, now entitled “Synchronized volcanism of the Mascarene Islands reveals short-period (~400 kyr) cyclic melt supply from the Réunion plume”, uses new and literature K-Ar ages, mapping, DEM’s and geomorphology assessment to constrain an updated chrono-stratigraphic framework and eruption frequencies through time, mostly Pleistocene, of the youngest edifices from the Reunion hotspot (Mauritius and Reunion islands). Their main finding is that both island volcanoes build up in similar time-frames of magma generation, and with a few hundred k-year intervals.

One of the main contributions of this work is the analysis and filtering of hundreds (if not thousands) of previous age data for both islands, but especially for Réunion, including their review, processing, and organization. I have no doubt that this work is a significant contribution to our understanding of both islands from several perspectives: their cyclical nature, periods of repose, and the possible coordinated functioning of both volcanic islands through a mantle hotspot.

After a thorough first revision, I had carefully read the new revised version of the manuscript, the response letter, the supplemental files and the changes throughout the document, including tables and figures and sections like methods and discussion. In my opinion, the authors addressed most of the points raised, at least by my revision and they cleared out most of my concerns. I see that they also did a really good job regarding the other reviewers’ comments.

The manuscript is now a much better version than the original one, and I consider that it is in good shape for the next step towards acceptance.

I’ll be happy to revise a new version in case it's necessary .

Best Regards,

Pablo Dávila-Harris

Reviewer #2

(Remarks to the Author)
Following the second review of the manuscript by Famin et al. on synchronized volcanism in the Mascarene Islands, I support its publication. The study presents a valuable integration of geochronological, field, and geomorphological data that significantly improves our understanding of hot-spot volcanism and temporal variations in magma supply.
However, in my opinion, some limitations remain that should be taken into account in future work, which does not preclude the publication of these results at this time. In particular, the interpretation of a recurrence interval of ~400 kyr requires caution. This estimate, derived from the spacing between reconstructed volcanic phases over the last ~3 Ma, shows a wide

range (370 ± 202 kyr) and is subject to uncertainties related to stratigraphic reconstruction, age constraints, and preservation bias. Therefore, it is best interpreted as a general trend toward episodic activity rather than as a robust periodic signal.
Despite these limitations, the identification of discrete and partially synchronous volcanic phases in Réunion and Mauritius provides strong evidence for mantle-controlled processes and supports the recurrent clustering of volcanic activity on timescales shorter than one million years. These aspects represent a significant contribution and provide a good direction for future studies aimed at refining our understanding of short-term variability in mantle plume dynamics.
Finally, the inclusion of a conceptual model illustrating the proposed mantle processes would further strengthen the manuscript, although this remains a suggestion rather than a requirement.

Reviewer #3

(Remarks to the Author)
The authors have carefully addressed the comments of three reviewers and provided a very detailed answer to each comment made. Although each reviewer focused on different elements of the manuscripts, the authors managed to address most point in a satisfactory;
Regarding my two main comments, the authors have added a significant new paragraph in the discussion acknowledging the limitations of the stratigraphic evidence used and providing solid argument to support their interpretation. They have also nuanced the interpretation of 'repose/inactive' periods.
I believe that the manuscript now present a solid piece of research based on well presented data, including extensive stratigraphic, geochronological and geomorphological constraints. Although the intepretation in terms of mantle plume cyclicity is still opened for discussion, I consider that this study is a great contribution to this scientific debate and should be published.

Open Access This Peer Review File is licensed under a Creative Commons Attribution 4.0 International License, which permits use, sharing, adaptation, distribution and reproduction in any medium or format, as long as you give appropriate credit to the original author(s) and the source, provide a link to the Creative Commons license, and indicate if changes were made.
In cases where reviewers are anonymous, credit should be given to 'Anonymous Referee' and the source.
The images or other third party material in this Peer Review File are included in the article’s Creative Commons license, unless indicated otherwise in a credit line to the material. If material is not included in the article’s Creative Commons license and your intended use is not permitted by statutory regulation or exceeds the permitted use, you will need to obtain permission directly from the copyright holder.
To view a copy of this license, visit https://creativecommons.org/licenses/by/4.0/

REBUTTAL LETTER

Synchronized volcanism of the Mascarene Islands reveals short-period (~400 kyr) cyclic melt supply from the Réunion plume

In the following letter, the reviewers' comments are reproduced in black, and our responses are provided in blue. Line numbers refer to the revised manuscript with tracked changes visible, unless otherwise specified.

Revised manuscript length:
Abstract: 146 words (instead of 145)
Main text without caption: 4216 words (instead of 4199)
Methods: 623 words (instead of 777)
Bibliography: 64 references (instead of 71)
7 Figures

Reviewer #1 (Remarks to the Author):

Validity
Although the data was filtered, analyzed, organized and plotted against their own, the study has some limited original data, but makes a good use of the available voluminous data set in terms of geochronology.
We emphasize that the ten new K–Ar dates presented here were not arbitrary additions to the ~400 previously published ages for Réunion and Mauritius, but were strategically selected to constrain key stratigraphic boundaries, specifically the bases and tops of lava-flow units and major erosion surfaces identified through several years of detailed fieldwork. We clarified this by mentioning that "[…] ten new radiometric ages by the unspiked Cassignol-Gillot K-Ar technique strategically selected to constrain key stratigraphic boundaries (see Methods)" (lines 193-194). These targeted constraints were critical for refining the chronostratigraphic framework of both islands. More broadly, the originality of this study lies not only in the acquisition of new ages, but in the integrated synthesis of geomorphology, stratigraphy, and a rigorously filtered geochronological dataset, which together reveal a coherent temporal pattern that had not previously been recognised.

The document provides field evidence, using DEM's, maps, drill-core data and geomorphological interpretations. As well as some field photos in the supplemental file. I found, however, that the general use of the geomorphological features is limited and not used in a thorough way. The authors only describe landscape aspects in a general form, not very concise and lacking some thorough revision of these features.
We thank the reviewer for this important comment. We have substantially revised the sentences dealing with geomorphology, to make the descriptions more precise and better integrated into the stratigraphic reasoning (see responses to specific comments). In particular, geomorphological terminology (e.g., unconformity types, inverted topography, erosion surfaces, valley incision patterns) has been clarified and explicitly defined at first occurrence. We have also modified landscape descriptions to focus on diagnostic criteria directly supporting the chronostratigraphic interpretation. In addition, the Methods section has been expanded to explain more clearly how

geomorphological observations were used in conjunction with radiometric ages and field mapping to identify constructional and erosional phases (lines 566-571). A Figure S11 has been added in the Supplementary Material to illustrate how geomorphology supports our stratigraphic subdivision in deduced from fieldwork and geochronology.

Significance

The findings are important and significant for an audience interested in hotspot volcanism, ocean island volcanism, magma production, evolution and periodicity. The main finding (relatively short 400 kyr eruption periods/clusters) do not contradict previous studies or puts it in conflict. However, the data available do not provide a very direct link to these observations.

We have clarified the connection between observations (field data, ages, and geomorphology), their direct interpretation (chronostratigraphies), and their geodynamic implications (plume-derived synchronization). In particular, the revised Discussion now explains more clearly how revised the revised chronostratigraphies lead to the recognition of a synchronized volcanism, interpreted as occurring in the mantle, possibly related to melt generation and melt delivery processes, as detailed in our responses below.

Data and methods

The manuscript contains three main sets of data: 1) field data; 2) geomorphological assessment; 3) K-Ar geochronology, published and performed by the authors.

Field data is well presented in terms of geological (lithological) maps of the islands, generalized stratigraphic columns, and cross sections in DEM's and drill core data logs. The geomorphological interpretations come from DEM's, one with 5 m resolution but the other with 30 m resolution, which is relatively low resolution to provide good geomorphological interpretations in such area. And finally, the K-Ar data: these is a robust data set, but mostly from the literature, it has been filtered and processed for quality control and correspond to around 400 data points organized in kyrs at a one sigma confidence level. However, if we compare this, with the original data, only 10 data points, it's relatively small.

About DEM resolution, see our response to the related specific comment below. About the limited number of new ages, we reiterate that the ten new ages were strategically targeted to constrain key stratigraphic boundaries. They provide essential temporal brackets that were previously lacking. The work presented here reflects extensive geological mapping and field investigation over both islands; the ten new ages represent the critical missing constraints needed to refine the chronostratigraphic framework.

The figures and tables are well presented in general, with some specific comments directly to them to improve the readability.

We thank the reviewer for the detailed comments onto the pdf file, which greatly helped us in our revision. These specific comments have been addressed as detailed below, and the figures have been improved accordingly.

Analytical approach

The analytical method used for the K.Ar age determinations looks thorough and comprehensive, but this applies mostly for their own samples and performed dates.

The problem is the control over these other 300 samples used to complete the model proposed, where they didn't have the control on those samples.

We thank the reviewer for raising this important point. Approximately 52% of the retained published and new ages (see statistics in the table below) were obtained at Géosciences Paris-Saclay (GEOPS) using the unspiked Cassignol–Gillot K–Ar technique, with rigorous sample selection and preparation procedures identical to those applied in this study. About 11% of the literature data were obtained using the $^{40}Ar/^{39}Ar$ method, for which plateau–isochron consistency provides strong quality control. An additional 6% consist of U–Pb, (U–Th)/He, or calibrated ^{14}C ages produced by our team in a previous paper, which are fully consistent with K–Ar ages[1].

The remaining 31% correspond to earlier whole-rock K–Ar ages. These were subjected to our stringent filtering procedure. Samples described as altered were systematically excluded. Ages with large uncertainties were removed too, which typically occurs when the samples are heterogeneous (due to xenolith and alteration phases, or because of interaction with atmospheric argon). All rejection criteria are documented in Table S2. In total, we have direct methodological control over 69% of the dataset, and the 31% remaining data were retained only after careful evaluation.
The Methods have been revised to:
(1) emphasize the advantages of the Cassignol-Gillot K-Ar dating method (for our new ages and the published ages from Table S2), lines 582-596;
(2) Better explain our stringent filtering procedure for ages, lines 625-641.

Dating technique	Piton des Neiges, data selected for interpretation	Piton de la Fournaise, data selected for interpretation	Mauritius, data selected for interpretation	Percent of retained data
U-Pb zircon, or (U-Th)/He zircon or apatite	4			6%
14C calibrated	15	2		
Ar/Ar respecting quality criteria	18		21	11%
K-Ar Cassignol-Gillot (this study, our team, and/or made in the same lab)	132	38	10	52%
K-Ar whole-rock validated based on sample description and/or cross-comparison with other methods	58	8	45	31%
Rejected data	30	4	59	

The geological approach is relevant, but the geomorphological approach is the one that is not very well developed throughout the document. Some of the geomorphological interpretations seem slightly vague and general. For example, in the use of concepts like "inverted topography", unconformities, breccias, sediments, scarps, paleosols, and other features that could be much better described/interpreted to align to the findings, or not.

The geomorphological interpretation has been clarified throughout the manuscript. In particular, terms like "unconformity" or "breccia" are now specified as "angular unconformities" (lines 269, 307; Fig. 4 caption), "sedimentary breccias" (lines 230, 569. Geomorphological terms like « inverted topography » are now defined at their first occurrence (lines 272-276).

Suggested improvements

General overview

• The geomorphological approach is the one that in my opinion is less well developed and it lacks a precise and ordered scheme. They use DEM's but at different resolutions, include some terms but used in a relatively vague manner.
See our responses to the related specific comments.

• The text is well written but somehow difficult to follow, mostly because the use of too many acronyms, several ages that interrupt the flow of the text, and complex sample numbers between sentences and phrases.
Several acronyms have been removed (e.g., LLSVP, CIR). Ages and sample names are now placed in brackets where possible to improve readability. However, we retain sample identifiers in the main text to allow careful cross-checking of observations. In addition, English correction has reduced the text and improved the readability.

• The conclusions reached could be supported by the data, but mostly the geochronology, not much support from the geomorphological approach in this aspect.
We agree that geochronology and field data are the primary drivers of our interpretation. Geomorphology is used to constrain spatial relationships rather than to define temporal phases independently. We have clarified this point in the Methods, lines 566-571 (see also response to the related specific comments).
We have also expanded the Discussion to include a paragraph explicitly addressing stratigraphic resolution and preservation bias (lines 422-435). This additional paragraph is compensated by English correction, leading to a main text length nearly identical to the first submission (4216 words instead of 4199).

• At the end, the discussions refer mostly to deep mantle magma dynamics, using several published studies and coupling it with their interpretations. This is fair, but the discussion should also refer to the other aspects worked in the manuscript like the geology/stratigraphy and geomorphology.
We have revised the Discussion to better integrate the geological, stratigraphic, and geomorphological constraints that underpin our chronostratigraphic reconstruction, rather than focusing exclusively on mantle dynamics. As said above, we now include an explicit paragraph addressing stratigraphic resolution, preservation bias, and the spatial representativeness of the identified volcanic phases (lines 422-435). We also better explain how synchronized volcanism over islands separated by 230 km lead us to suggest a mantle process (lines 449-463). These additions clarify how the deep-mantle interpretations are grounded in field-based observations. While the geodynamic implications remain a central objective of the study, the revised text more clearly emphasizes the geological and geomorphological foundations of our conclusions.

Specific points:

- A pdf is attached with specific points that require attention to improve the quality of the manuscript.
All the comments made onto the pdf have been addressed, as detailed below.

- Although not distracting from conclusions, the written manuscript could be improved by the revision of a native English speaker/writer.
The manuscript has been edited for proper English, which results in a contraction of the text compensating the added paragraphs.

- The title is short and could appear slightly misleading or not informative enough. I would suggest including more information without extending it too much.
The title has been revised to improve clarity while remaining concise:
“Synchronized volcanism of the Mascarene Islands reveals short-period (~400 kyr) cyclic melt supply from the Réunion plume.”

Responses to comments on the attached pdf files :

Title: Again, the title has been revised.

About geomorphology (previously line 90), « The "geomorphology" part of the manuscript is, in my opinion, not very well developed. I would suggest to be more specific in terms of which type of morphological features enabled to make a better set of conclusions/interpretations, related to the magma/melt production of this volcanism. »: The geomorphological component of the manuscript has been substantially strengthened as follows:
(1) Again, the term ‘inverted topography’ is now explicitly defined at its first occurrence (lines 272-276).
(2) Unconformity types, volcaniclastic breccias, and sedimentary units are now described using more precise terminology and are illustrated in Figures 4, S9, and S10. The corresponding figures 3, 4, S4, S9, and S10 and their captions have been revised to reflect this clarified nomenclature.
(3) Erosion contrasts are more thoroughly explained in the main text (e.g., lines 242–243) and are supported by additional visual documentation in Figures 4 and S11.
(4) A new Supplementary Figure (Fig. S11) presents elevation profiles illustrating slope changes and erosion contrasts between stratigraphic units on the 30-m DEM of Mauritius. This figure demonstrates that meaningful geomorphological interpretation remains possible despite the relatively low DEM resolution.
(5) The Methods section now provides a clearer explanation of how slope breaks and erosion contrasts are used to identify distinct stratigraphic units (lines 568-571), and explicitly discusses the limitations inherent to this geomorphological approach (lines 571–574).

Figure 1: font size has been increased (11 pt instead of 8 pt) to improve readability.

« degenerative » (previously line 120): replaced by « post-shield » (line 163).

About the 30-m DEM used for Mauritius (previously line 140): « This is not a very high resolution DEM. To me it's Ok, but 30, you most likely miss several geomorphological [features ?] »
We totally agree with this comment. The only publicly available DEM for Mauritius is the 30 m SRTM, and we would certainly be much more precise in the contours of our units with a better DEM. However, DEM resolution only affects the contours of our stratigraphic units, not their subdivision. As said above, we added a new Figure S11

with elevation profiles, showing that the SRTM of Mauritius, despite its low resolution, can be used to observe contrasted patterns in slope erosion corresponding to the different units identified with field observations and clusters of ages (as also displayed in Figures 5, S6, and S8).

Line 155: « I notice that the geological ages in the document are defined as ka, even when the ages reach the Ma number. I understand this is a matter of format or stylñe that the authors have decided and I'm ok with that. However, I would suggest a short section to justify the use of this nomenclature. In other words, when you have a 2847 +/- 41 ka, is more common to use the Ma format: 2.847 +/- 0.41 Ma. If the Editor and the journal guidelines agree to leave this format, it is fine for me. ».
Ages and durations are expressed consistently in ka and kyr throughout the manuscript to maintain uniform numerical precision and to match the temporal resolution of our dataset (~10 kyr). Using Ma for older ages would require switching units within the same chronostratigraphic framework, while younger Quaternary ages would need to be expressed to three decimal places (e.g., 0.027 ± 0.001 Ma), reducing clarity and internal consistency. We also believe that switching from Ma to ka back and forth would alter readability. We have now added a sentence to the Methods section to clarify this choice (lines 641-643)

About Figure 2: a) « white line is too thin here in this picture. Include a relative scale », and b) « No scale in this panel. ». We added a horizontal scale bar for each panel. The two pictures and the 3D DEM views are displayed without any vertical exaggeration, so the horizontal bar also applies to the vertical scale. In all 3D DEM views (including in the Supplementary Material, i.e., Figs 2-5, S4-S6, and S8-S10), we added a scale bar, together with a caption sentence: « Scale bars apply to the foreground; scale varies with perspective.”

About the nature of the “sediments (breccia)” in Figure 2’s caption: “Please be clear. Sediments or breccia, and if both, what type of breccias. »: We have replaced the original wording with “volcaniclastic sedimentary breccias” to better reflect the observed characteristics (lines 230-231). A more specific interpretation (e.g., lahar deposits, debris-avalanche deposits, or slope/fluvial volcaniclastic deposits) is not possible at this stage, as the outcrop shown in Figure 2 is located mid-cliff and can only be examined at a distance using binoculars. From this vantage point, the breccia appears unsorted and matrix-supported, with clasts of variable size and both rounded and angular fragments. We therefore prefer to retain a descriptive and non-genetic terminology to avoid overinterpretation.

About published ages (formerly lines 180 and 185): “I think this does not needs to by specified. When referencing the paper would be enough to define that the date you are referring to is from the literature. If those are publicly available dates, counts as data, and it can and should be used in order to improve our knowledge. ».
« Published » has been removed (line 239).

Formerly line 188: « Define type of method for this age » (age of sample 69H4 in Kluska, 1997): Dating methods for all the literature ages are already reported in Table S2. Adding the methods for each literature age would considerably expand the main text and alter readability. We left this sentence unchanged.

About Figure 3: « This general use of the term « sediments » for undifferentiated (holocene/quaternary?) deposits could perhaps be modified. Sediments is a broad term (includes sand, gravel, etc). ».
We agree that this point needs clarification. In the context of our geological maps, the term “sediments” encompasses a range of unconsolidated deposits, including fluvial cobbles, gravels, and sands from the Bras de la Plaine River, as well as lahars, debris-avalanche breccias, slope-derived volcaniclastic deposits in the cirque of Cilaos, and even coral sands in Mauritius. Because these deposits are not systematically differentiated at the mapping scale adopted in this study, a more detailed classification cannot be applied consistently across the map. To improve clarity, we have replaced the generic term “sediments” with “volcaniclastic sediments” in Fig. 3, but we could not change the other figures, which include too many types of sediments.

What's the age of the volcanic cones? Are all younger that 200 ka?
Most of the volcanic cones shown on the map have not been directly dated, and we therefore cannot assign precise ages to them. However, these cones display very limited erosion and well-preserved morphologies, indicating that they are likely among the youngest volcanic features on the island. On this basis, and by comparison with dated units, we infer that they are most probably younger than ~200 ka, which justifies their placement above the PF3–4 unit in the legend. We have clarified this point by adding this sentence in the caption: “Volcanic cones, overlying the other constructional units, are attributed to the PF3-4 period.” (lines 263-264).

In Figure 3’s caption, about the log: « If its a drill core, why you have two different points for sample 22RE01 and 22RE05? Shouldn't be the same locality? ».
Thank you for this comment, this is indeed a label mistake. The samples 22RE01 and 22RE05 have been collected at different locations, and only the latter was collected in the core. Figure 3’s caption has been corrected accordingly (line 261).

About the term « inverted topography » (formerly lines 207, 239) : « If this is a recurrent geomorphological feature in the island, I suggest to expand slightly more, or perhaps include a figure at the supplemental."
In the first occurrence, we now define explicitly the stratigraphic implication of this geometry (lines 272-276).

About Figure 5: « I wonder if any of these scarps could be landslide-related? No mention of ocean island landslide in the long history of both massive volcanoes, is there any landslide/mass wasting process event identified before? This could be relevant to the hiatus/no deposition or unconformity interpretations. »
We thank the reviewer for this pertinent remark. Indeed, Dyment et al. (2012)[2] suggested that a major collapse occurred on the western flank of Mauritius at ~3.5 Ma, based on large-scale geomorphological asymmetry and linked it to rejuvenated volcanism. However, subsequent seismic reflection and bathymetric surveys (e.g., Lebas, PhD thesis[3]) did not confirm the presence of large offshore landslide deposits consistent with such a catastrophic event.
Importantly, Figure 5 focuses on southern Mauritius, which would not have been directly affected by the western-flank collapse proposed by Dyment et al[2]. The

unconformities discussed in this figure are supported by stratigraphic relationships and radiometric age constraints observed locally.
We acknowledge that smaller-scale landslides and mass-wasting processes are likely to have occurred during the island's evolution, as is common for ocean island volcanoes. However, there is currently no evidence that the specific hiatuses and unconformities described in this study are related to large-scale flank collapse events.

Please revise this phrase (formerly llines 279-281) : "The fresh basaltic flow...".
The sentence has been revised (lines 361-364).

Formerly line 287 : « Please, include evidence of this observations [geomorphological arguments (slope changes, erosion patterns)], even in the supplemental file, if they are important elements for the interpretation of hiatuses. »
As said above, a new Figure S11 has been added to the Supplementary Material, presenting representative elevation profiles that illustrate slope contrasts and differential erosion between units YS3 (380–200 ka) and YS4 (≤200 ka) of the Younger Series in Mauritius. These profiles provide quantitative support for the geomorphological criteria used to identify hiatuses.
Together with Figures 5, S6, and S8, Figure S11 demonstrates that slope breaks and erosion contrasts between chronostratigraphic units remain discernible despite the relatively coarse (30 m) DEM resolution available for Mauritius.

Figure 6 : This figure is very important, however, its size and amount of information is overwhelming to really understand the output. I would suggest to either make it larger or remove certain elements. For example, the geological maps or panel C),. If possible, make two figures from this one or split some information in another existing figure.
Panel C has been moved to the Supplementary Materials as a new Figure S12. Figure 6 becomes larger and easier to read.

Formerly lines 319-320: This [the match between PN2 and YS2] is a good example, clearly defined with the ages in the text, but the figure is too small to perceive.
As the former panel c) has been moved to the Supplementary Material, two figures (6 and S12) are now called to support this sentence (line 395).

Formerly lines 330: I see isotopic data but not chemical data. If not provided, please modify this phrase and refer only to isotopic data or refer to a literature source.
We have added a supplementary Table S4 with a compilation of major and trace element data about the Mascarene Islands, as well as two new graphics (now in Figure S13) showing that Réunion and Mauritius also display different trace element ratios as a function of time, suggesting different melting sources and/or ascent processes. The caption has been modified accordingly. The isotopic database becomes Table S5.

About the apex of the rising plume (formerly lines 365-366), « Refer to figure and show example based on data or literature ».
The sentence has been modified for proper English, and now adds two references and a figure citation (Figs 7, S13) about the apex of the plume above Réunion (lines 483-488).

About porosity and permeability (formerly line 381), « These two terms should be briefly introduced for the use in mantle melt dynamics, because their conventional use in geology is at another scale, orders of magnitude smaller. »
We have modified the two corresponding sentences to define the porosity and permeability waves in a mantle context (lines 508-514).

About Figure 7 : «Enlarge the depth scale text, teh percentage and isolines. »: Done.
« What are these black vertical lines? »: They correspond to the vertical length of plume quanta (~60 km) in the hypothesis of a plume pulsation. This is now explained in Figure 7's caption (line 548).

About porosity waves in Figure 7: This concept should be referred and better explained in the text. I understand it provides from previous studies, but if used as a strong argument in the model, should be better described.
The concept of « porosity waves » is not a strong argument in the model, it is just one of the possible interpretations for the periodic volcanism. This concept was already described lines 390-395, so we modified Figure 7 and its caption to highlight the link with the explanation of the main text (lines 549-550).

About the term « pancake » in the caption : « This term is used several times in the text. I would suggest to define its use briefly somewhere. If extracted from a source, please be clear. Why a "pancake" form? Why not a globe or a funnel or just a tabular shape? If extracted form seismic tomography interpretations, this should be specified.
Analog and numerical models of plume/lithosphere interaction all predict that plumes spread at the base of the lithosphere due to viscosity contrasts, not as globes or funnels and nor as perfectly tabular shapes, but as gradually flattening bodies. Grifith and Campbell[4] are arguably the first to describe spreading plume heads as "pancakes" in a publication, but it is Ribe and Christensen[5] who popularized the use of this term. This term is now widely used in Nature publications, without further definition (e.g., Ballmer et al.[6]). In our previous version of the manuscript, the plume pancake was already defined on its first occurrence, but we modified the sentence, added the most famous reference that popularized the use of this word, quotation marks, and a call to Figure 7 to be more specific about « pancakes » (lines 477-481).

Methods : What type of breccias? Please be specific. Lavas can include autobreccias or carapace breccias that not precisely mean hiatus or no deposition periods that can be used as unconformities.
Thank you for this comment. The breccias used in this study are all of sedimentary origin. We added the term « sedimentary breccia horizons» to be more specific (line 569).

This is a very complete explanation of the K-Ar method used in this study, however, in my opinion is far to extent for this section. I would recommend synthesize for the most important steps and the rest pass into the supplemental file.
We agree that the original description of the K–Ar analytical procedure was too detailed for the main Methods section. At the same time, clarification of the methodological framework was necessary to address reviewer #2's concerns about the apparent paradox between our new ages and published data. To accommodate both points, we have transferred the detailed description of the Cassignol–Gillot

analytical protocol to the Supplementary Material, while retaining in the main Methods section a concise explanation of the rationale for selecting the K–Ar technique and its relevance to this study. These modifications have reduced the length of the Methods (623 words instead of 777) and the number of references (64 instead of 71).

Reviewer #2 (Remarks to the Author):

In summary, this paper presents a rich and well-argued case for short-period magmatic pulsation in the Réunion hotspot and its implications for mantle plume dynamics. However, additional clarification of the methodology, broader context through comparison with other hotspots (particularly Hawaii), and careful distinction between generation and migration of melts would substantially strengthen the manuscript.
As explained below, the geochronological methodology has been clarified (with the K-Ar protocols moved to the Supplementary Material), a supplementary Table S6 has been added for comparison with other hotspots. The comparison with Hawaii is only proposed as a future work for a broader implication of 400 kyr periodicities in hotspots (lines 557-558).

GENERAL COMMENTS.
1. The concluding suggestion that approximately 400 kyr magmatic pulsation may be a global feature is intriguing but underdeveloped. The case of Hawaii, where similar datasets (such as dozens to hundreds of high-precision U-Th/He and Ar-Ar ages) exist, is not discussed. Are there similar periodicities observed there, or is Réunion truly unique? Without this comparison, the claim of global relevance remains speculative.
We did not intend to claim global applicability of a ~400 kyr periodicity. The revised text now frames this as an open question rather than a conclusion (lines 552-562). As explained above, we added a Supplementary Table S6 compiling 200–400 kyr activity phases reported from other Indian Ocean intraplate islands. We did not include Hawaii because a proper evaluation would require a comprehensive, standalone study given the volume of available data. The final paragraph has been rewritten accordingly (lines 557-562).

2. In lines 70-71: The authors refer to the Deccan Traps as a suspected cause of the Cretaceous–Paleogene mass extinction, citing voluminous flood basalt eruptions. However, the role of the Chicxulub impact as a primary or at least co-contributing factor in the extinction event is widely accepted. The authors should acknowledge the complexity of this debate and clarify that current consensus generally considers a combination of both impact and volcanism as drivers of the extinction, rather than attributing it solely to Deccan volcanism.
We agree that the phrasing was overly simplistic. The revised text now cites, with an added reference, Deccan volcanism and the Chicxulub impact as major drivers of the Cretaceous-Paleogene transition (lines 100-102).

3. In line 133, the manuscript notes that some units, such as Rodrigues, are constrained only by two K-Ar ages published before the development of modern dating techniques. However, the present study also relies primarily on the K-Ar

method. This raises a conceptual inconsistency: if earlier K-Ar ages are considered outdated, why was the same technique chosen for new data acquisition? The authors justify their use of K-Ar for young mafic rocks, but it remains unclear why more advanced or complementary techniques, such as Ar-Ar, U-Pb, or U-Th/He, were not employed. Given the emphasis on high-resolution temporal patterns, a discussion of the methodological limitations and advantages of K-Ar compared to modern alternatives is warranted.

In fact, there is no conceptual inconsistency, because the K-Ar technique applied in this study has tremendously evolved since the early methodology of K-Ar dating, with the following improvements:

(1) The early K-Ar technique, and in particular the two ages for Rodrigues published in 1965, were made using K-Ar on whole rock, before knowing that alteration, xenocrysts, reheating etc. could corrupt the data. The unspiked Cassignol-Gillot technique resolves this problem by using carefully separated phases such as groundmass or plagioclase microcrysts. This precaution ensures that only the latest solidified magmatic phases are used for dating.

(2) The historical and modern K–Ar techniques rely on fundamentally different analytical procedures. Early K–Ar dating was based on isotope dilution, whereby the ^{40}Ar signal (measured in volts or amperes) was converted into absolute ^{40}Ar atoms through the addition of a calibrated ^{38}Ar spike. The introduction of this spike limited analytical precision, particularly for young samples with low radiogenic argon contents.

In contrast, the unspiked Cassignol–Gillot K–Ar method employs a highly stable mass spectrometer and determines the ^{40}Ar signal by comparison with a known atmospheric ^{40}Ar reference, thereby avoiding the addition of a ^{38}Ar spike and improving precision for young, low-K basalts.

Furthermore, advances in low-current electrometers, ultra-high-vacuum systems, and mass spectrometer electronics have substantially enhanced analytical stability and sensitivity compared with instruments available several decades ago.

The modern unspiked Cassignol-Gillot K-Ar technique is as advanced as the Ar-Ar or (U-Th/He) methods, and generally yields similar or even better uncertainties for rocks younger than 1 Ma.

Lines 582-596, we have clarified:

- The analytical improvements of modern K–Ar methods (lines 582-585)
- Differences from early whole-rock approaches (lines 585-587)
- The limitations of ^40Ar/^39Ar for very young, low-K basalts (lines 587-591)
- The restricted applicability of U–Pb and (U–Th)/He methods to basaltic systems (lines 591-596)

4. While the observed synchrony of volcanic activity between Réunion and Mauritius is interesting, the interpretation that this pattern necessarily reflects periodic melt generation deep in the mantle plume may be premature. Alternative explanation, such as episodic magma storage and release, could also produce a similar volcanic signal. The authors are encouraged to clarify whether the ~400 kyr periodicity reflects the timing of melt generation at depth or rather the dynamics of melt transport and eruption. A clearer distinction between these processes: melt generation versus melt delivery, would strengthen the interpretation of the underlying geodynamic mechanism.

We agree this distinction is essential. The Discussion now explicitly:
(1) Explains why a mantle-scale process is required (given synchronization across 230 km and across a heterogeneous lithosphere), lines 448-463.
(2) Separates two hypotheses: Periodic melt generation within the plume, or cyclic melt extraction/transport, lines 489-491.
(3) Discusses separately the two different hypotheses, plume pulsation (lines 495-507) or melt transport (lines 508-518).

5. The authors cite general references to 200–400 kyr activity phases in other intraplate volcanic settings, but this should be expanded into a more substantive comparison, possibly as a table or extended figure in the supplementary material, to strengthen the broader relevance of the findings.
We only formulated the comparison with other intraplate volcanic systems as a question. But we understand that we need to both strengthen the comparison and add caution to its possible broader implication. In the revised manuscript, we have expanded the comparison by compiling reported 200–400 kyr-long phases of volcanic activity and repose from other intraplate volcanic islands from the Indian Ocean into a new Supplementary Table (Table S6). This table summarizes the volcanic setting, dating methods, temporal resolution, and duration of reported activity phases for each case (with the same format as Table S3).
We emphasize at the end of the revised Discussion that, although such durations have been described in several systems, they have generally not been evaluated for periodic behaviour or synchronization across multiple edifices (lines 554-559). The inclusion of Table S6 therefore strengthens the comparative framework while clearly distinguishing previously reported phase durations from the cyclic and island-scale synchronization documented here.

Reviewer #3 (Remarks to the Author):

I have two related remarks that could be considered by the authors when revising their publication:

- The authors described a few geological sections in which several phases of volcanic activity are clearly separated by erosion discontinuities. Based on similar dates for the early and late lava flows of each phase at these different locations, they make the fundamental hypothesis that the two islands evolved through island-wide constructional phases separated by island-wide erosional phases. This hypothesis is mentioned explicitly on line 258 ‘considering these erosion events as island-wide episodes’, but is made implicitly throughout. If one consider the current evolution of Piton de la Fournaise, we see active accumulation of lava flows over the last decades/centuries in some sectors, with concomitant development of deep erosional canyons in others. This suggests, as observed on other volcanoes where topography or deformation concentrate eruptive products on some flanks, that constructional and erosional processes can be intimately intertwined on volcanoes and that differentiation might be more spatial than temporal (especially on large shields for which eruptive activity can concentrate in some sectors due to stress field evolution). While I understand that the locations where stratigraphic evidence can be studied are limited by the availability of sections created by erosion, authors should further discuss their rational for assuming that the sequences (both phases of activity and

erosion) documented are representative for the entire island. And if there are, is it possible to reconstruct the approximate morphology of the island at these different stage of evolution, in order to estimate the volume of magma associated with each phase?

This is a very thoughtful comment. We indeed make the fundamental assumption that lava units, discontinuities, and clusters of radiometric ages (or hiatuses of ages) observed over several cross sections can be extrapolated to constructional phases and erosion periods within an entire volcano. We agree that stratigraphic reconstructions of volcanic islands are inherently conditioned by exposure and preservation, and that constructional and erosional processes may be spatially decoupled on large shield volcanoes. Stratigraphy in general, including in volcanic systems, necessarily relies on the number and distribution of observable outcrops and cross-sections, and correlations may be influenced by lateral discontinuity, topographic control on lava emplacement, and erosion-driven incompleteness of the record. These caveats are widely recognized in volcanic stratigraphic methodology[7] and in studies of stratigraphic completeness[8].

There is, however, a methodological advantage in working in Quaternary volcanoes rather than in older terranes: we can leverage observed eroded slopes to check the spatial repartition of the lava units and erosion intervals recognized in cross sections and dated by radiometric techniques.

Considering the example of Piton des Neiges, this methodology is very robust for PN4 to PN1a periods (backward in time) and for erosion periods in between, because these units are observed radially on all the slopes of the volcano, with multiple cross sections, and with very contrasted erosion patterns for the different units (e.g., Fig. S2). The slopes of the LM unit are observed only in the north of Piton des Neiges, but angular unconformities are found in many places at the bottom of the Cilaos and Salazie cirques, supporting the assumption that the LM phase, and erosion after it, took place all over the volcano. We admit that the existence of the LM0 phase, and the erosion that followed before LM, rely on a single cross section in the Bras de Saint Suzanne river, north of the Mafate cirque. Is the assumption of an island-wide erosion after PN0 justified in this case? The low elevation of the top of the PN0 unit (<350 m above sea level) suggests that Piton des Neiges (and Réunion Island) was barely emerging at that stage, and thus that the volume of landscape subject to erosion was limited too, lending credit to the hypothesis that the observed erosion discontinuity affected the totality of the (small) emerged PN0 unit. Nevertheless, we acknowledge that our assumption of volcano-wide construction and erosion episodes looses power and resolution toward the oldest units of Réunion and Mauritius islands (see also our response to the next comment).

The current evolution of Piton de la Fournaise is a good example to further test our hypothesis of observation completeness, because indeed, canyons are incised while eruptions take place at the same time. We note that all the eroded canyons (Rivière des Remparts, Bras de la Plaine, Rivière de l'Est, Langevin, Basse Vallée) are invaded by very young (<10 ka) lava flows (reported in the geological map), making it perfectly possible to identify that the volcano is currently active, even in the most actively eroded zones. This supports the idea that, at the scale of 50 kyr and over, active volcanoes radially spread deposits all over their slopes, and that we do not « miss »major construction or erosion episodes, even though structures certainly channels eruptions and incision.

In the revised Discussion we have added a paragraph, to explicitly acknowledge that the volcanic phases identified here represent periods of enhanced or reduced volcanic output at the island scale rather than complete island-wide shutdowns (lines 424-426). We also emphasize that our interpretation of synchronized cyclicity does not rely on a single outcrop or unconformity, but on the repeated coincidence of radiometric age clusters and erosion surfaces across spatially independent sections on two islands (lines 431-435). This combination of independent chronostratigraphic constraints reduces the likelihood that the identified phases result solely from local geomorphic effects.

About reconstructing the volumes associated with each phase: We agree that reconstructing palaeo-morphologies and estimating eruptive volumes for each constructional phase would provide valuable quantitative constraints on magma output. However, such estimates would require full three-dimensional reconstruction of the volcanic edifices, knowing the geometry of eroded units that have now been buried by more recent units.
Our objective here is to resolve the temporal organization of volcanic activity and repose at sub-Myr resolution, rather than to quantify precise volumetric fluxes. Because large portions of the older edifices have been removed by erosion or are not exposed, any phase-by-phase volume calculation based solely on preserved outcrops would carry substantial and difficult-to-quantify uncertainties. We have therefore clarified in the revised manuscript that the identified phases are interpreted as intervals of enhanced or reduced volcanic output, without attempting detailed volumetric reconstruction. Future work integrating geophysical data and 3D geomorphological modelling would be required to address this question robustly (lines 437-439).

- Associated with the previous point, figure 6 highlights that the period of repose between the different phases of activity tend to decrease with time, with hiatus of less than 50.000 years between the 3 phases of activity defined over the last million years at the three volcanoes considered. One would wonder if these more limited evidence of absence of activity in the recent period cannot be linked to the fact that recent volcanic deposits are outcropping over more areas of the islands, have been less affected by erosion and have been assessed by more dating. By consequence, we could question whether the discrete phases of activity separated by long hiatus for the period over 1Myr are not largely due to the limited outcropping of these phases, the destruction of some deposits by erosion and the limited number of available dating.
I do not mean that the main point made by the authors is not valid, but these limitations, which are intrinsic to the study of the geological stratigraphy of volcanoes based on field evidence, should be acknowledge and discussed by authors. Authors could also consider whether the pulsation of the plume should be leading to full cessation of volcanic activity or rather to fluctuation of the volumetric output rate over time, and whether this volumetric output rate through time can be reconstructed based on geological evidence (especially for the most recent phases with more widespread outcrops).
We totally agree with the reviewer that the apparent duration of activity repose intervals increases backward in time, and suggest a potential influence of preservation and sampling bias on our stratigraphies. In the added paragraph, we now explicitly acknowledge that stratigraphic and geochronological resolution

decreases backward in time due to erosion, burial, and lower dating density (lines 426-428). We clarify that the shorter apparent hiatuses in younger successions may partly reflect improved preservation and sampling (lines 428-430). However, we also emphasize that the recurrent ~400 kyr cyclicity emerges from age clustering and hiatuses observed independently on two islands, suggesting that the signal cannot be solely attributed to preservation bias (lines 431-437). We further clarify that the identified phases are interpreted as fluctuations in magma supply rather than complete island-wide cessation of activity. The added paragraph addresses the question of the completeness of stratigraphic reconstruction, the temporal bias in stratigraphic record, and the volume reconstructions. The Methods also exposes the underlying assumption of stratigraphic reconstruction based on multiple observations (lines 571-574).

Minor comments:
- Line 88 : 'two latest volcanic edifices'. Authors refer to each island as one volcanic edifices, whereas Reunion Island is build from at least two volcanoes (PN and PF). Terminology used should be clarified.
Indeed, we took care to call islands as the emerged parts of « volcanic edifices », which are made of one or more volcanoes. In the case of the volcanic edifice of Réunion Island, there are two volcanoes, PN and PF. This terminology has been consistently applied all-over the manuscript, leading to four corrections (lines 82, 107, 124, and 150).

- Lines 178-187 : dates reported in that paragraph do not match the age limits of the classes used in figure 3. Especially for base of PN1b, the date of 1116 is conflicting with the limit of 1113 ka. If there is a reason for these differences, it should be explained.
The base of PN1b is constrained by two K-Ar ages: our age of 1116 ± 20 ka (detailed in text), and a published age of 1110 ± 20 ka. Because the uncertainties overlap, we took the weighted average of these two data as the lower limit of PN1b unit, i.e. 1113 ± 20 ka. We have revised Table S3 to better explain this: « Base of the PN1b unit at 1116 ± 20 ka (22RE05, this study, Figs 2b, S5) or 1110 ± 20 ka (RU9 and RU16 in McDougall, 1971). The beginning is estimated as the weighted average of these two ages (cell I13 in Table S3).

References cited in the rebuttal letter

1. Famin, V. *et al.* Multitechnique Geochronology of Intrusive and Explosive Activity on Piton des Neiges Volcano, Réunion Island. *Geochem Geophys Geosyst* **23**, (2022).
2. Dyment, J. *et al. A Large, Catastrophic Flank Collapse in Mauritius ~3.5 Millions Years Ago?* (2012).
3. Lebas, E. Processus de démantèlement des édifices volcaniques au cours de leur évolution : application à La Réunion et à Montserrat et comparaison avec d'autres édifices. (2012).
4. Griffiths, R. W. & Campbell, I. H. Stirring and structure in mantle starting plumes. *Earth and Planetary Science Letters* **99**, 66–78 (1990).
5. Ribe, N. M. & Christensen, U. R. The dynamical origin of Hawaiian volcanism. *Earth and Planetary Science Letters* **171**, 517–531 (1999).

6. Ballmer, M. D., Ito, G., Van Hunen, J. & Tackley, P. J. Spatial and temporal variability in Hawaiian hotspot volcanism induced by small-scale convection. *Nature Geosci* **4**, 457–460 (2011).
7. Martí, J., Groppelli, G. & Brum da Silveira, A. Volcanic stratigraphy: A review. *Journal of Volcanology and Geothermal Research* **357**, 68–91 (2018).
8. Sadler, P. M. Sediment Accumulation Rates and the Completeness of Stratigraphic Sections. *The Journal of Geology* **89**, 569–584 (1981).

Point-by-point response to the reviewers' comments

Reviewer #1 (Remarks to the Author):

The revised manuscript, now entitled "Synchronized volcanism of the Mascarene Islands reveals short-period (~400 kyr) cyclic melt supply from the Réunion plume", uses new and literature K-Ar ages, mapping, DEM's and geomorphology assessment to constrain an updated chrono-stratigraphic framework and eruption frequencies through time, mostly Pleistocene, of the youngest edifices from the Reunion hotspot (Mauritius and Reunion islands). Their main finding is that both island volcanoes build up in similar time-frames of magma generation, and with a few hundred k-year intervals.
One of the main contributions of this work is the analysis and filtering of hundreds (if not thousands) of previous age data for both islands, but especially for Réunion, including their review, processing, and organization. I have no doubt that this work is a significant contribution to our understanding of both islands from several perspectives: their cyclical nature, periods of repose, and the possible coordinated functioning of both volcanic islands through a mantle hotspot.
After a thorough first revision, I had carefully read the new revised version of the manuscript, the response letter, the supplemental files and the changes throughout the document, including tables and figures and sections like methods and discussion. In my opinion, the authors addressed most of the points raised, at least by my revision and they cleared out most of my concerns. I see that they also did a really good job regarding the other reviewers' comments.
The manuscript is now a much better version than the original one, and I consider that it is in good shape for the next step towards acceptance.
I'll be happy to revise a new version in case it's necessary .
Best Regards,
Pablo Dávila-Harris

Our response: We sincerely thank Pablo Dávila-Harris for this thorough, constructive, and positive evaluation of our revised manuscript. We greatly appreciate his recognition of the significance of our dataset compilation and filtering, as well as the improved clarity and robustness of the revised version.

We are pleased that the reviewer considers that we have satisfactorily addressed the concerns raised during the first round of review, and that the manuscript is now suitable for the next stage toward acceptance.

Following his first comment of increasing the clarity of the title, we have made a final adjustment to the manuscript title to comply with the journal requirement of fewer than 15 words. The revised title now reads: *"Synchronized Mascarene volcanism reveals 400 kyr cycles in melt supply from the Réunion plume."*

We have also added the reviewer's name to the Acknowledgements

Reviewer #2 (Remarks to the Author):

Following the second review of the manuscript by Famin et al. on synchronized volcanism in the Mascarene Islands, I support its publication. The study presents a valuable integration of geochronological, field, and geomorphological data that significantly improves our understanding of hot-spot volcanism and temporal variations in magma supply.
However, in my opinion, some limitations remain that should be taken into account in future work, which does not preclude the publication of these results at this time. In particular, the interpretation of a recurrence interval of ~400 kyr requires caution. This estimate, derived from the spacing between reconstructed volcanic phases over the last ~3 Ma, shows a wide range (370 ± 202 kyr) and is subject to uncertainties related to stratigraphic reconstruction, age constraints, and preservation bias. Therefore, it is best interpreted as a general trend toward episodic activity rather than as a robust periodic signal. Despite these limitations, the identification of discrete and partially synchronous volcanic phases in Réunion and Mauritius provides strong evidence for mantle-controlled processes and supports the recurrent clustering of volcanic activity on timescales shorter than one million years. These aspects represent a significant contribution and provide a good direction for future studies aimed at refining our understanding of short-term variability in mantle plume dynamics.

Our response: We sincerely thank Yamirka Rojas-Agramonte for this thoughtful and balanced assessment of our manuscript, and for highlighting both its contributions and remaining limitations.

We fully agree that the ~400 kyr recurrence interval should be interpreted with caution. As noted by the reviewer, the uncertainty is substantial (370 ± 202 kyr), reflecting limitations in stratigraphic reconstruction, age constraints, and preservation bias—particularly for volcanic phases older than ~1 Ma (e.g., PN0, LM, and PN1), where temporal resolution is reduced.

We considered alternative approaches, such as weighting the uncertainty on volcanic phases by age to account for the loss of temporal resolution back in time. However, we chose to retain a conservative, non-weighted estimate of uncertainty in recurrence intervals, in order to avoid introducing additional assumptions into the analysis.

Given the magnitude of the uncertainty (approximately 54% of the mean recurrence interval), we agree that the signal is more appropriately interpreted as reflecting a general tendency toward episodic, clustered volcanic activity rather than a strictly periodic behavior. In line with this, we have replaced the term "periodic" with "quasi-periodic" in our former revision.

While this comment does not require further modification of the manuscript at this stage, we agree that additional statistical analyses and improved geochronological constraints will be essential to better resolve the temporal structure of melt supply in future work.

We thank the Yamirka Rojas-Agramonte again for these insightful comments, which have helped to clarify the interpretation and strengthen the manuscript.

Finally, the inclusion of a conceptual model illustrating the proposed mantle processes would further strengthen the manuscript, although this remains a suggestion rather than a requirement.

Our response: A conceptual model illustrating the proposed mantle processes is already presented in Figure 7 (captioned "Conceptual model") in the manuscript. This figure was designed to synthesize our interpretation of the temporal and spatial relationships between volcanic phases in Réunion and Mauritius, and their link to variations in melt supply from the mantle plume.

In light of the reviewer's comment, we carefully reconsidered whether further additions or modifications were needed. We conclude that Figure 7 adequately captures the conceptual framework of the study, and therefore no changes have been made to the manuscript in this regard.

Reviewer #3 (Remarks to the Author):

The authors have carefully addressed the comments of three reviewers and provided a very detailed answer to each comment made. Although each reviewer focused on different elements of the manuscripts, the authors managed to address most point in a satisfactory;
Regarding my two main comments, the authors have added a significant new paragraph in the discussion acknowledging the limitations of the stratigraphic evidence used and providing solid argument to support their interpretation. They have also nuanced the interpretation of 'repose/inactive' periods.
I believe that the manuscript now present a solid piece of research based on well presented data, including extensive stratigraphic, geochronological and geomorphological constraints. Although the intepretation in terms of mantle plume cyclicity is still opened for discussion, I consider that this study is a great contribution to this scientific debate and should be published.

Our response: We sincerely thank the reviewer for this careful and constructive evaluation of our revised manuscript, and for the time devoted to both rounds of review.

We greatly appreciate the reviewer's recognition of the improvements made in response to the initial comments, particularly the addition of a new discussion paragraph addressing the limitations of the stratigraphic framework and the refinement of our interpretation of "repose" or "inactive" periods.

We agree that the interpretation of the observed cyclicity in terms of mantle plume processes remains open to discussion. We have therefore aimed to present this interpretation in a balanced and cautious manner, emphasizing both its implications and its current limitations.

We thank the reviewer again for supporting the publication of this work.
